# A draft genome assembly of the Chinese sillago (*Sillago sinica*), the first reference genome for Sillaginidae fishes

**DOI:** 10.1093/gigascience/giy108

**Published:** 2018-09-10

**Authors:** Shengyong Xu, Shijun Xiao, Shilin Zhu, Xiaofei Zeng, Jing Luo, Jiaqi Liu, Tianxiang Gao, Nansheng Chen

**Affiliations:** 1 Fishery College, Zhejiang Ocean University, Zhoushan, Zhejiang316022, China; 2 Wuhan Frasergen Bioinformatics Co., Ltd., Wuhan, Hubei 430075, China; 3 School of Life Sciences, Yunnan University, Kunming, Yunnan 650500, China; 4 CAS Key laboratory of Marine Ecology and Environmental Sciences, Institute of Oceanology, Chinese Academy of Sciences, Qingdao, Shandong 266071, China; 5 Laboratoryfor Marine Ecology and Environmental Science, Qingdao National Laboratory for Marine Science and Technology, Qingdao, Shandong 266237, China; 6 Department of Molecular Biology and Biochemistry, Simon Fraser University, Burnaby, Canada

**Keywords:** Sillaginidae, Chinese sillago, PacBio sequencing, Canu, FALCON, genetic diversification

## Abstract

**Background:**

Sillaginidae, also known as smelt-whitings, is a family of benthic coastal marine fishes in the Indo-West Pacific that have high ecological and economic importance. Many Sillaginidae species, including the Chinese sillago (*Sillago sinica*), have been recently described in China, providing valuable material to analyze genetic diversification of the family Sillaginidae. Here, we constructed a reference genome for the Chinese sillago, with the aim to set up a platform for comparative analysis of all species in this family.

**Findings:**

Using the single-molecule real-time DNA sequencing platform Pacific Biosciences (PacBio) Sequel, we generated ∼27.3 Gb genomic DNA sequences for the Chinese sillago. We reconstructed a genome assembly of 534 Mb using a strategy that takes advantage of complementary strengths of two genome assembly programs, Canu and FALCON. The genome size was consistent with the estimated genome size based on *k*-mer analysis. The assembled genome consisted of 802 contigs with a contig N50 length of 2.6 Mb. We annotated 22,122 protein-coding genes in the Chinese sillago genomes using a *de novo* method as well as RNA sequencing data and homologies to other teleosts. According to the phylogenetic analysis using protein-coding genes, the Chinese sillago is closely related to *Larimichthys crocea* and *Dicentrarchus labrax* and diverged from their ancestor around 69.5–82.6 million years ago.

**Conclusions:**

Using long reads generated with PacBio sequencing technology, we have built a draft genome assembly for the Chinese sillago, which is the first reference genome for Sillaginidae species. This genome assembly sets a stage for comparative analysis of the diversification and adaptation of fishes in Sillaginidae.

## Data Description

**Figure 1:**
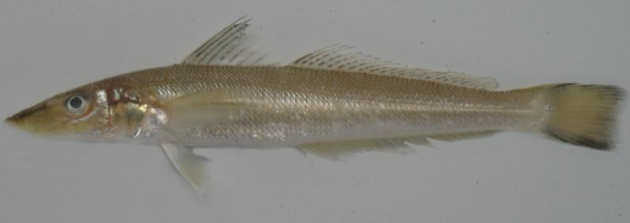
A representative individual of the Chinese sillago.

The fish family Sillaginidae consists of demersal marine fishes commonly known as sand whitings or sand borers [1] that inhabit inshore waters throughout the Indo-West Pacific [2, 3]. As ecologically and commercially important marine organisms, Sillaginidae species play important roles in the commercial fisheries of Pakistan, Australia, China, Malaysia, Thailand, and the Philippines [1, 4]. Owing to similar phenotypic characteristics, delineation and identification of Sillaginidae species often confuse the taxonomists [5, 6]. Additionally, rapid environmental changes resulting from anthropogenic activities can force Sillaginidae species to adapt to diversifying situations, leading to further diversification and speciation [7, 8]. Numerous cryptic lineages were identified in *Sillago sihama* complex by using phenotypic traits and molecular markers in the Northwestern Pacific [9]. For example, five recently identified sillago species were misidentified as *S. sihama* solely using phenotypic data [5, 6, 10-12]. Therefore, it is essential to investigate Sillaginidae species at the genetic level to identify molecular features for accurate characterization of different species and for understanding rapid genetic diversification and speciation. Using a combined method with morphological and phylogenetic analysis of the mitochondrial DNA cytochrome oxidase subunit I gene, the Chinese sillago, *Sillago sinica* (Fig. 1, Fishbase ID: 65964), is one of the most recently identified Sillaginidae species in the Northwestern Pacific [5]. Due to their phenotypic similarity, *S. sinica* was previously misidentified as *S. sihama* [5]. However, these two fish species are different because *S. sinica* inhabits a cold-temperate environment, while *S. sihama* inhabits a warm-temperate environment [9]. It is thus essential to sequence the genome of *S. sinica*, which will improve taxonomy and may help to reveal insights into evolutionary history of Sillaginidae species and the role of environmental changes in rapid genetic diversification and speciation [5, 6, 13, 14].

Here, we present a reference genome assembly for *S. sinica* constructed using long reads generated by the Pacific Biosciences (PacBio) DNA sequencing platform Sequel and using a genome assembly strategy that takes advantage of two genome assemblers, Canu [15] and FALCON [16]. This genome assembly of the Chinese sillago is the first genome constructed for the family Sillaginidae. The completeness and continuity of the genome provide valuable genomic resources for studies on the evolutionary history of the rapid speciation processes of Sillaginidae species.

### Sample and DNA extraction

To obtain enough genomic DNA for the PacBio Sequel platform (Pacific Biosciences of California, Menlo Park, CA, USA), we collected fresh epaxial white muscle tissue from a Chinese sillago fish in Zhoushan City, Zhejiang Province. The sample was quickly frozen in liquid nitrogen for 1 hour before storing at −80°C. Genomic DNA was extracted using a standard phenol/chloroform extraction protocol. The integrity of genomic DNA molecules was checked using agarose gel electrophoresis, showing a main band around 20 kb and satisfying the requirement for PacBio library construction by the manufacturer's protocol.

### Genome size estimation

To estimate the Chinese sillago genome size, we also sequenced the genomic DNA using Illumina next-generation sequencing (NGS) technologies. Four paired-end libraries with insert sizes of 250 bp, 300 bp, 500 bp, and 800 bp and one mate-pair 2-kb library were constructed from 20 μg DNA molecules. Then, 35, 42, 31, 39, and 18 Gb of data were generated, representing the genome coverage of 67X, 81X, 60X, 75X, and 35X for 250 bp, 300 bp, 500 bp, 800 bp, and 2 kb, respectively, resulting in ∼165.5 Gb NGS data (a coverage of ∼317X) (Table 1, Supplementary Table S1) on the Illumina HiSeq X Ten platform (Illumina Inc., San Diego, CA, USA).

**Table 1:** Summary of sequence data from *Sillago sinica*.

Type	Method	Library size (bp)	Data size (Gb)	Read N50 (bp)
DNA	HiSeq X Ten	250	34.8	150
		300	42.2	
		500	31.4	
		800	38.8	
		2000	18.3	
DNA	PacBio Sequel	20,000	27.3	12,957
RNA	HiSeq X Ten	250	10.5	150

The sequencing data used in this work. Note that read N50 length for the PacBio Sequel were measured for subreads.

The quality of raw reads was evaluated using FastQC (FastQC, RRID:SCR_014583) [17] and then filtered by quality and length using HTQC [18]. Low-quality bases and reads were filtered in the following filtering steps with FastQC and HTQC: (1) removing adaptor sequences introduced during sequencing library construction; (2) removing read pairs if the average base quality was lower than 20 for any of the two ends; (3) trimming ambiguous or low-quality fragments at two ends of reads within a window size of 5 bp and an average quality threshold of 20; and (4) removing read pairs if any of the two reads had a read length shorter than 75. Using FastQC for cleaned sequencing reads, a single peak around 45% was identified in Guanine-Cytosine (GC) distribution (Supplementary Fig. S1). Next, 10,000 read pairs were randomly selected and searched against a nonredundant nucleotide (nt) database with the Basic Local Alignment Search Tool-Nucleotide (BLASTN) tool [19]. We found that the best hits of reads were enriched for closely related fish species [20], including medaka (*Oryzias latipes)*, large yellow croaker (*Larimichthys corcea*), common carp (*Cyprinus carpio*), seabass (*Dicentrarchus labrax*), and zebrafish (*Danio rerio*) (Supplementary Table S2), indicating no obvious contamination was observed in the sequencing data.

By analyzing the 17-mer depth distribution from the 300-bp library cleaned sequencing reads in gce software [21], we estimated the genome size of the Chinese sillago using the following equation:

\begin{equation*}
{\rm{G}} = {{\rm{N}}_{17{\rm{{-}mer}}}}/{{\rm{D}}_{17{\rm{ - mer}}}}
\end{equation*}

where the N_17–mer_ was the total number of 17-mers, D_17_–_mer_ denoted the peak frequency of 17-mers estimated, and G represented the estimated genome size. The N_17–mer_ was 37,811,957,476 in our data, and D_17–mer_ was estimated as 66 in gce software [21], suggesting the coverage of sequencing data for the Chinese sillago genome was about 66 and an estimated genome size of 524 Mb according to the above equation. We also used the *k*-mer of 21 and 27 for the analysis and found that the estimated genome size ranged from 519 to 524 Mb (Supplementary Table S3). Meanwhile, we observed a heterozygous and a repeat peak (Supplementary Fig. S2) with an estimated heterozygosity of 0.66%–0.76% (6.6–7.6 single nucleotide polymorphism (SNP)/1000 nt) and a repeat content of 11.3%–12.7% for the Chinese sillago, according to the statistical model in gce software [21]. The heterozygosity of our sample was noticeably higher than for other fish species in previous genome studies [22-24], partly because the Chinese sillago sample used in this project was collected directly from the wild environment without further artificial inbreeding. Many artificial breeding techniques in aquaculture, such as inbreeding and gynogenesis, could effectively decrease the genomic heterozygosity and potentially reduce the difficulty of the genome assembly [24].

Using short reads with various insert lengths, we performed a pilot assembly solely with NGS data. Genomic heterozygosity is one of the biggest challenges of many complex genome assemblies, and the Platanus (Platanus, RRID:SCR_015531) package was designed for heterozygous genome assembly [25]. Therefore, the Platanus package [25] with the default parameters was applied for our pilot genome assembly. As a result, we constructed a 624-Mb genome assembly with more than 1 million contigs and a contig N50 length of 3.2 kb (Table 2).

**Table 2:** Genome assembly statistics for *Sillago sinica*.

Method	Type	Genome size (Mb)	Longest sequence (Mb)	Sequence number	Sequence N50 (Mb)
Platanus	contig	624	0.091	1,045,226	0.0032
	scaffold	518	0.735	187,308	0.042
FALCON	contig	546	7.8	2,066	1.50
Canu	contig	527	7.4	1,349	1.62
Final	contig	534	9.2	802	2.60

The assembly is the result of using various methods and sequencing data. Note that Platanus were used for NGS data assembly, and FALCON and Canu were used for PacBio data.

### Genome assembly with long PacBio reads

The pilot assembly with traditional short sequencing data resulted in a highly fragmented assembly reference genome for Chinese sillago, especially on the continuity of contig level. Previous studies illuminate the excellent performance of PacBio long reads on complex genome assembly [26, 27]. We therefore applied PacBio to generate long reads, aiming to generate a longer contig assembly for the genome. To this end, we prepared two 20-kb genomic DNA libraries, which were sequenced using PacBio Sequel with five single molecule real-time (SMRT) cells, generating 27.3 Gb raw DNA reads with a genomic coverage of ∼53 X (Table 1, Supplementary Table S4). After removing adaptor sequences, we obtained 3.4 million subreads (total, 27.2 Gb) with a read N50 length of 12.96 kb (Supplementary Table S5, Supplementary Fig. S3).

**Figure 2:**
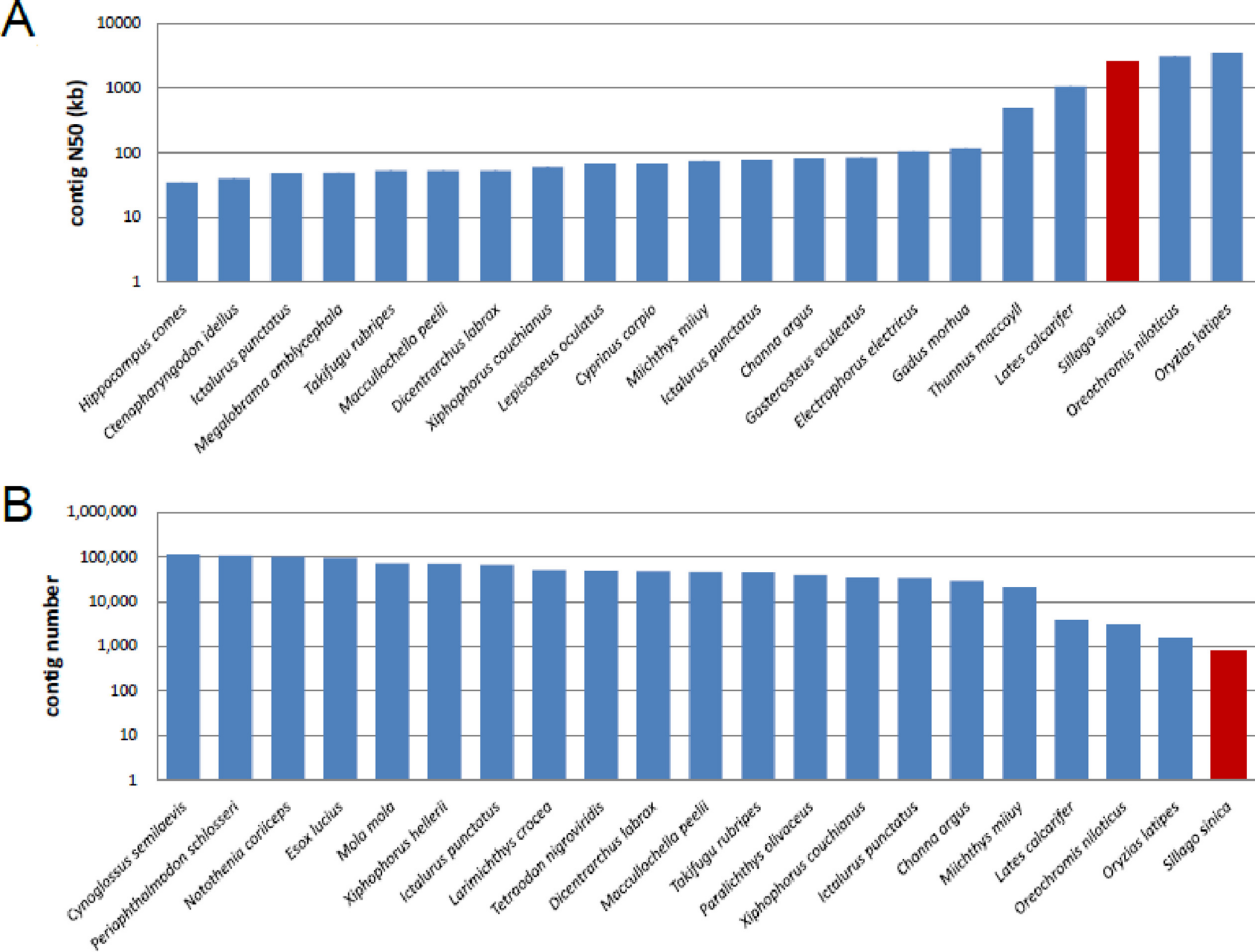
Comparing genome assemblies between Chinese sillago and other fish species. The *y*-axis represents the contig N50 **(A)** and contig number **(B)**. Only the top 20 public genomes are shown (*x*-axis) ordered by contig N50 length **(A)** and contig number **(B)**.

Because of the high heterozygosity for the Chinese sillago, we first used FALCON (FALCON, RRID:SCR_016089) [16] for genome assembly. With the parameter of length_cutoff set at 10 kb and pr_length_cutoff at 8 kb, we produced a 546-Mb genome assembly for the Chinese sillago, which agreed well with the estimated genome size in 17-mer analysis (see above). The genome assembly consisted of 2,066 contig with a N50 length of 1.5 Mb (Table 2). Meanwhile, we also applied Canu [15] v1.4 (Canu, RRID:SCR_015880) to assemble the genome with the CorrectedErrorRate parameter set at 0.052. As a result, we obtained a second Chinese sillago genome of 527 Mb, with 1,349 contigs and contig N50 of 1.62 Mb (Table 2). Thus, both assemblies have similar genome sizes and excellent continuity. We then used Genome Puzzle Master (GPM) [28] to merge the two genome assemblies into an integrated genome by tracking the overlapping relationships between contigs of the two genome assemblies and applied Redundans [29] (v0.13c) to remove the sequence redundancy. The resulting genome assembly was further polished using NGS data, which were used in the genome survey analysis above. The contig N50 length of the final 534-Mb Chinese sillago genome assembly reached 2.6 Mb (Table 2). The contig N50 of the Chinese sillago was much higher than those of previous fish genome assemblies constructed using NGS DNA sequencing technologies and was comparable with those of recently reported model fish species [27, 30] (Fig. 2).

### Genome quality evaluation

To validate the completeness of the Chinese sillago genome assembly, we subjected the sequences to Core Eukaryotic Genes Mapping Approach (CEGMA) (CEGMA, RRID:SCR_015055) [31] and Benchmarking Universal Single-Copy Orthologs (BUSCO) (BUSCO, RRID:SCR_015008) [32] evaluation. More than 96% of core eukaryotic genes were successfully identified in the Chinese sillago genome in both CEGMA (Supplementary Table S6) and BUSCO (Supplementary Table S7) analyses, and more than 92.8% were detected as complete single-copy BUSCO genes, implying a high completeness of the Chinese sillago genome assembly.

To further evaluate the accuracy of the Chinese sillago genome assembly, we aligned the NGS-based short reads from whole-genome sequencing data against the genome assembly using the Burrows–Wheeler aligner (BWA, RRID:SCR_010910) [33]. We found that 98.4% of the reads were reliably aligned to the genome assembly, and 95.8% of the reads were properly aligned to the genome with their mates. The insertion length distribution for sequencing libraries of 250 bp, 300 bp, 500 bp, 800 bp, and 2 kb exhibited a single peak around the sequencing library length chosen (Supplementary Fig. S4), illuminating the high quality of the genome assembly. Using genomic homozygous mutations detected from the NGS data, we estimated that the genome accuracy at the base level reached 99.997%.

### Repeat annotation

We annotated repetitive elements in the Chinese sillago genome using Tandem Repeat Finder [34]. To identify transposon elements (TE), RepeatModeler (RepeatModeler, RRID:SCR_015027) was used to identify *de novo* repeat types in the genome. The Repbase database [35] of known repeats and a *de novo* repeat library generated by RepeatModeler were used. The TEs in the Chinese sillago genome were then identified by mapping to the library using the software RepeatMasker (RepeatMasker, RRID:SCR_012954) [36].

We found that tandem repeat content in Chinese sillago (4.69%) was much higher than the content in *Gasterosteus aculeatus* (2.03%), *Larimichthys corcea* (2.7%), *Oryzias latipes* (0.92%), and *Dicentrarchus labrax* (2.8%). However, the content of TEs (12.86%) of the Chinese sillago was lower than the content of the above-listed fish species (Supplementary Fig. S5, Supplementary Table S8), leading to an overall lower content of repetitive sequences in the Chinese sillago genome, which might be a reason for the relatively small genome size of Chinese sillago.

### RNA preparation and sequencing

Using Illumina sequencing technologies, we also sequenced cDNA libraries prepared from the same Chinese sillago individual fish used for genome annotation. Ocular, skin, muscle, gonadal, intestinal, liver, kidney, blood, gallbladder, and air bladder tissues were collected, and RNAs were extracted with TRIZOL Reagent (Invitrogen, USA). RNAs were then balance mixed for the sequencing. The absorbance of 1.90 at 260 nm/280 nm and the RNA integrity number (RIN)of 9.1 were obtained for the purified RNA sample by Nanodrop ND-1000 spectrophotometer (LabTech, USA) and 2100 Bioanalyzer (Agilent Technologies, USA), respectively.

According to the protocol suggested by the manufacturer, 1 μg of RNA was reverse transcribed using the Clontech SMARTer cDNA synthesis kit and further fragmented using divalent cations for NGS sequencing. The paired-end library was prepared following the manual of the Paired-End Sample Preparation Kit (Illumina Inc., San Diego, CA, USA). Finally, the library with an insert length of 300 bp was sequenced by Illumina HiSeq X Ten in 150PE mode (Illumina Inc.). As a result, we obtained ∼10.4 Gb transcriptome data from RNA sequencing (Table 1, Supplementary Table S1).

### Gene and functional annotation

To annotate genes in the Chinese sillago genome, gene prediction was performed with *de novo*, homology-based, and transcriptome sequencing-based methods. We first used Augustus (Augustus: Gene Prediction, RRID:SCR_008417) [37] to predict protein-coding genes in the Chinese sillago genome. Then, protein sequences of closely related fish species, including *Danio rerio, Dicentrarchus labrax, Gasterosteus aculeatus, Larimichthys corcea, Oryzias latipes, Takifugu rubripes, and Gadus morhua*, were downloaded from Ensembl [20] and aligned against the Chinese sillago genome using TBLASTN software [38]. GeneWise (GeneWise, RRID:SCR_015054) [39] was then used to define gene models. We also used NGS transcriptome short reads aligned on the Chinese sillago genome using the TopHat (TopHat, RRID:SCR_013035) package [40], and the gene structures were predicted using Cufflinks (Cufflinks, RRID:SCR_014597) [41]. All gene models were then integrated using MAKER [42] to obtain a consensus gene set (Supplementary Fig. S6). We annotated 22,122 protein-coding genes in the Chinese sillago genome. The gene number, gene length distribution, coding sequence (CDS) length distribution, exon length distribution, and intron length distribution were comparable with those in other teleost fish species (Supplementary Fig. S7, Supplementary Table S9).

To obtain functional annotation of the protein-coding genes in the Chinese sillago genome, we searched the National Center for Biotechnology Information nonredundant protein (nr), nonredundant nucleotide (nt), and Swissprot database using local BLASTX and BLASTN programs with an e-value threshold of 1e-5 [19]. We then searched the Gene Ontology (GO) [43] and Kyoto Encyclopedia of Genes and Genomes (KEGG) [44] pathway databases using the software Blast2GO (Blast2GO, RRID:SCR_005828) [45]. As a result, most (21,768) of the 22,122 genes were annotated by at least one database, representing 98.4% of the total genes (Supplementary Fig. S8, Supplementary Table S10). We also annotated four types of noncoding RNAs (microRNAs, transfer RNAs, ribosomal RNAs, and small nuclear RNAs) using tRNAscan-SE (tRNAscan-SE, RRID:SCR_010835) [46] and the Rfam database [47] using Infernal (Infernal, RRID:SCR_011809) [48] (Supplementary Table S11).

### Gene family identification

**Figure 3:**
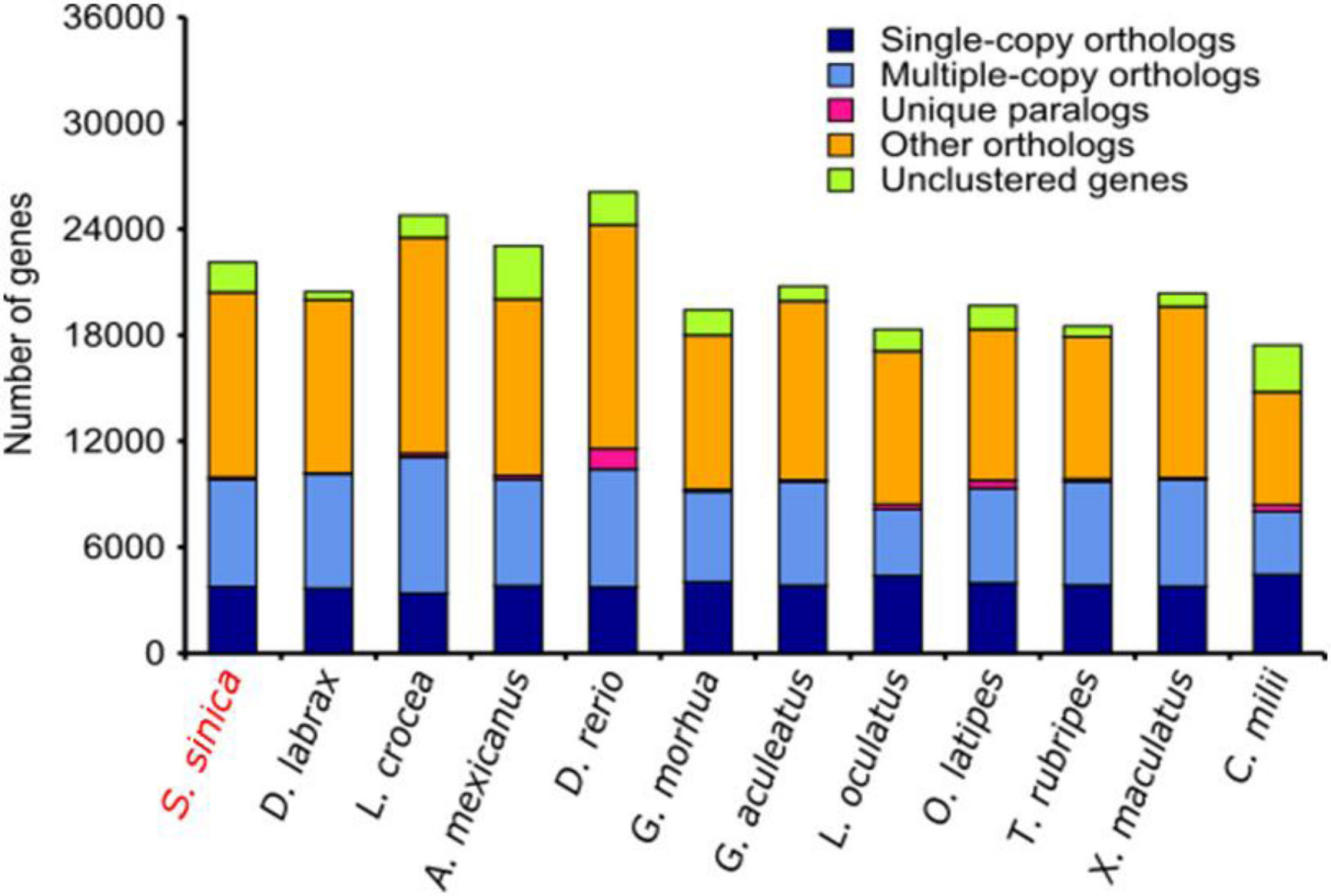
Gene family comparison between Chinese sillago and other fish species (*x*-axis). The *y*-axis represents the gene number for each class: single-copy (one gene for each species), multiple-copy (more than one gene for each species), unique paralogs (no genes in other species), other orthologs (other cases in gene clusters), and unclustered genes (genes that did not cluster with other genes).

In order to identify gene families among fish species, proteins of the longest transcripts of each individual genes from the Chinese sillago and other fish species, including *Dicentrarchus labrax, Larimichthys corcea, Astyanax mexicanus, Danio rerio, Gadus morhua, Gasterosteus aculeatus, Lepisosteus oculatus, Oryzias latipes, Takifugu rubripes, Xiphophorus maculates*, and *Callorhynchus milii*, were aligned to each other with BLASTP [19] programs with an e-value threshold of 1e-5. The high-scoring segment pair (HSP) segments were concatenated by Solar, and H-scores were calculated from Bit-score. Gene families were obtained by clustering of homologous gene sequences using H-scores in Hcluster_sg software. As a result, 15,022 gene families were constructed for the Chinese sillago (Fig. 3).

### Phylogenetic analysis for Chinese sillago and fishes with public genome

**Figure 4:**
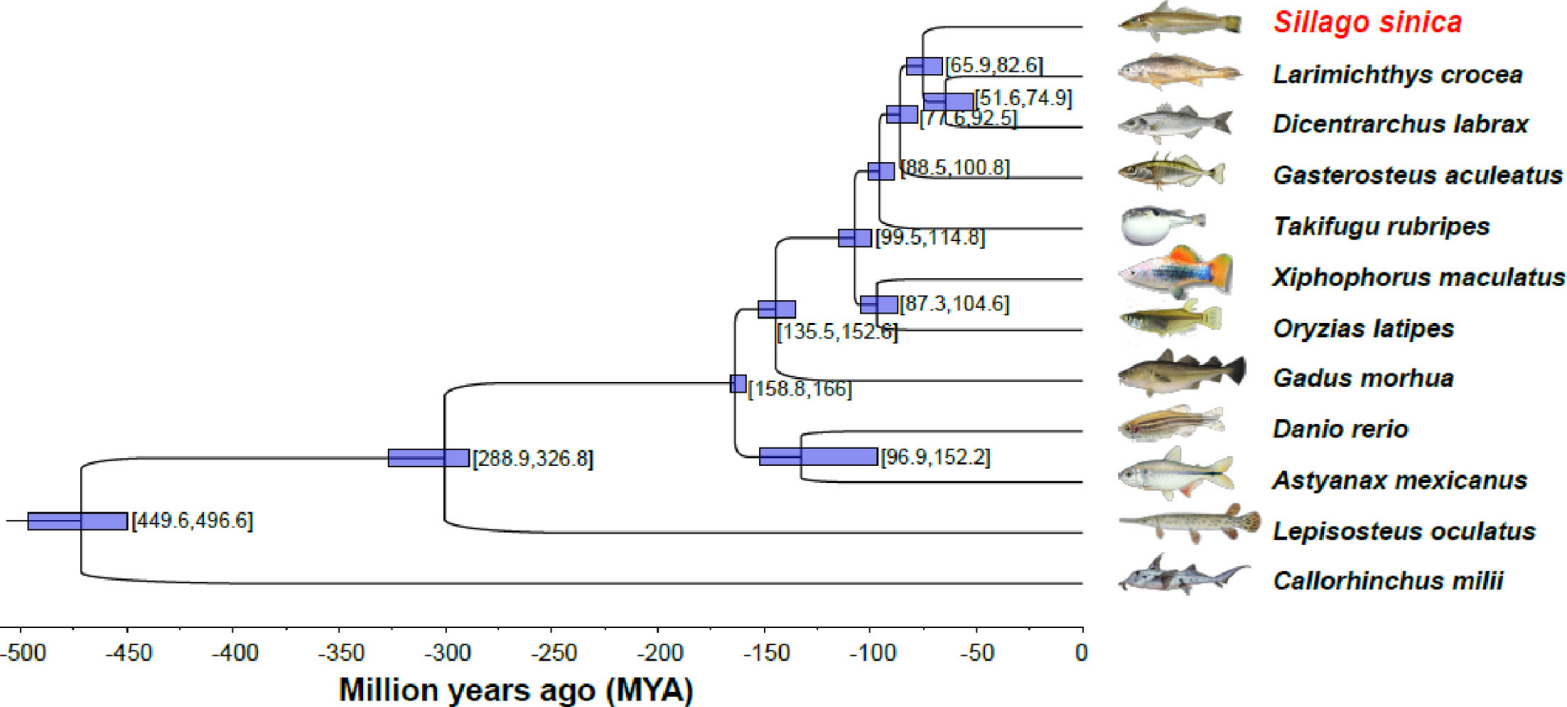
The phylogenetic relationship of Chinese sillago with other fish. The estimated divergence time (million years ago) is shown in the lower coordinates. The blue bars at each branch represent the 95% confidence interval of the species divergence time.

To generate the phylogenetic relationship of Chinese sillago with other fish species, the coding sequences of single-copy gene families among all species were extracted and aligned with the guidance of protein alignment from the ClustalW program [49], and the alignments were concatenated as a single dataset. The maximum-likelihood method implemented in the PhyML (PhyML, RRID:SCR_014629) package [50] with the JTT+G+F model was used to construct the phylogenetic tree from the super alignment of the coding sequences. The MCMCtree program in the PAML (PAML, RRID:SCR_014932) package was used to determine divergence times with the approximate likelihood method [51] and molecular clock data from the divergence time between zebrafish and medaka from the TimeTree database [52]. According to the phylogenetic analysis, Chinese sillago were clustered together with *Larimichthys crocea* and *Dicentrarchus labrax*, which was consistent with the fish species taxonomy. Chinese sillago diverged from the common ancestor with *Larimichthys crocea* and *Dicentrarchus labrax* around 69.5–82.6 million years ago (Fig. 4).

## Conclusion

Using long reads from the third-generation PacBio Sequel sequencing platform, we successfully assembled the genome of the Chinese sillago, which represents the first reference genome of all species in Sillaginidae. The 534-Mb Chinese sillago genome assembly consists of 802 contigs with contig N50 length of 2.6 Mb. The contig N50 is longer than those of most fish genome assemblies and is comparable with those of recently reported model fish species. The genome base-level accuracy reached 99.997%. We annotated 22,122 protein-coding genes in the Chinese sillago genome assembly. We found that Chinese sillago diverged from the common ancestor of *Larimichthys crocea* and *Dicentrarchus labrax* around 69.5–82.6 million years ago. The genome assembly, together with gene annotation and transcriptome data generated in this work, provide a valuable resource for research on the phylogenetic and adaption investigation of the Sillaginidae family.

## Availability of supporting data

Raw sequencing data are deposited in the Sequence Read Archive with accession number SRR6965224-SRR6965233. Supporting data and materials, also including the genome assembly and annotations, are available in the *GigaScience* GigaDB database [53].

## Additional files

**SI Figure S1**. GC and sequence distribution for *Sillago sinica* genome assembly.

**SI Figure S2**. 17-mer analysis for genome size estimation.

**SI Figure S3**. Polymerase length distribution from PacBio SEQUEL sequencing.

**SI Figure S4**. Genome sequence validation using NGS reads from libraries with various insertion length.

**SI Figure S5**. Sequence divergence rate of TEs in Chinese sillago against to RepBase.

**SI Figure S6**. Venn plot for gene prediction using different method.

**SI Figure S7**. Chinese sillago gene structure comparison to other teleost.

**SI Figure S8**. Functional annotation of genes predicted in the Chinese sillago genome.

**SI Table S1**. DNA and RNA sequencing for Chinese sillago based on NGS.

**SI Table S2**. Species hit table by searching NGS sequencing reads to the NCBI nt database.

**SI Table S3**. *K*mer-based method to estimate the genome characters.

**SI Table S4**. Polymerase statistics for DNA genome sequencing based on PacBio.

**SI Table S5**. Subread statistics for DNA genome sequencing based on PacBio.

**SI Table S6**. CEGMA result to analysis genome completeness for Chinese sillago.

**SI Table S7**. BUSCO result to analysis genome completeness for Chinese sillago.

**SI Table S8**. Repeat annotation in Chinese sillago genome.

**SI Table S9**. Protein-coding gene prediction in Chinese sillago genome.

**SI Table S10**. Functional annotation of predicted protein-coding genes.

**SI Table S11**. Non-coding gene prediction in Chinese sillago genome.

## Abbreviations

BLAST: Basic Local Alignment Search Tool; BUSCO: Benchmarking Universal Single-Copy Orthologs; BWA: Burrows–Wheeler aligner; CEGMA: Core Eukaryotic Genes Mapping Approach; GO: Gene Ontology; KEGG: Kyoto Encyclopedia of Genes and Genomes; NGS: next-generation sequencing; nt: nucleotide; PacBio: Pacific Biosciences; SNP: Single Nucleotide Polymorphism; TE: transposon element.

## Ethics statement

This study was approved by the Animal Care and Use Committee of Fishery College of Zhejiang Ocean University.

## Competing interests

The authors declare that they have no competing interests.

## Funding

This study was supported by a grant from the National Natural Science Foundation of China (41776171; 31572227; 31602207), the Scientific Startup Foundation of Zhejiang Ocean University (Q1505), the Open Foundation from Fishery Sciences in the First-Class Subjects of Zhejiang (No.20160001) and the CAS Pioneer Hundred Talents Program (to N.S.C.).

## Author contributions

T.X.G. and N.S.C. conceived the project. S.Y.X. collected the samples and extracted the genomic DNA. S.J.X., S.L.Z., X.F.Z., and J.Q.L. performed the genome assembly and data analysis. T.X.G., N.S.C., S.J.X., and J.L. wrote the paper.

## Supplementary Material

GIGA-D-18-00112_Original_Submission.pdf

GIGA-D-18-00112_Revision_1.pdf

Response_to_Reviewer_Comments_Original_Submission.pdf

Reviewer_1_Original_Submission_(attachment).pdf

Reviewer_1_Report_(Original_Submission) -- Larry Croft 4/15/2018 Reviewed

Reviewer_2_Report_(Original_Submission) -- Bastiaan Star 05/14/2018 Reviewed

Reviewer_3_Report_(Original_Submission) -- Julian Catchen 05/31/2018 Reviewed

Supplemental Files
