# Supplementary material for: A draft genome assembly of the Chinese sillago (Sillago sinica), the first reference genome for Sillaginidae fishes
Source: Gigascience. 2018 Sep 10;7(9):giy108. doi: 10.1093/gigascience/giy108 (PMC6143730; doi:10.1093/gigascience/giy108)
Supplement: GIGA-D-18-00112_Revision_1.pdf [file giy108_giga-d-18-00112_revision_1.pdf]

GigaScience

A draft genome assembly of the Chinese sillago (Sillago sinica), the first reference genome for Sillaginidae fishes

--Manuscript Draft--

**Manuscript Number:**	GIGA-D-18-00112R1
**Full Title:**	A draft genome assembly of the Chinese sillago (Sillago sinica), the first reference genome for Sillaginidae fishes
**Article Type:**	Data Note
**Funding Information:**	National Natural Science Foundation of China (CN) (41776171) — Dr tianxiang gao<br>National Natural Science Foundation of China (31572227) — Dr tianxiang gao<br>National Natural Science Foundation of China (CN) (31602207) — Dr Shijun Xiao<br>Scientific Startup Foundation of Zhejiang Ocean University (No. Q1505) — Dr tianxiang gao<br>the Open Foundation from Fishery Sciences in the First-Class Subjects of Zhejiang (No. 20160001) — Dr tianxiang gao
**Abstract:**	Background<br>Sillaginidae, also known as smelt-whitings, is a family of benthic coastal marine fishes in the Indo-West Pacific that have high ecological and economic importance. Many Sillaginidae species, including the Chinese sillago (Sillago sinica) are recently described in China, providing us with valuable materials to analyze genetic diversification of the family Sillaginidae. Herein, we constructed a reference genome for the Chinese sillago, with the aim to setup a platform for comparative analysis of all species in this family.<br>Findings<br>Using the single-molecule real-time DNA sequencing platform PacBio Sequel, we generated ~27.3 Gb genomic DNA sequences for the Chinese sillago. We reconstructed a genome assembly of 534 Mb using a strategy that taking advantage of complementary strengths of two genome assembly programs Canu and FALCON. The genome size was consistent with the estimated genome size based on Kmer analysis. The assembled genome consisted of 802 contigs with a contig N50 length of 2.6 Mb. We annotated 22,122 protein-coding genes in the Chinese sillago genomes using de novo method and with RNA-seq data and homologies to other teleosts. According to the phylogenetic analysis using protein-coding genes, Chinese sillago was closely related to Larimichthys Crocea and Dicentrarchus labrax, and Chinese sillago diverged from their ancestor around 69.5 - 82.6 million years ago.<br>Conclusions<br>Using long reads generated with PacBio sequencing technology, we have built a draft genome assembly for the Chinese sillago, which is the first reference genome for Sillaginidae species. This genome assembly sets a stage for comparative analysis of the diversification and adaptation of fishes in Sillaginidae.
**Corresponding Author:**	tianxiang gao<br><br>CHINA
**Corresponding Author Secondary Information:**	
**Corresponding Author's Institution:**	
**Corresponding Author's Secondary Institution:**	

Powered by Editorial Manager® and ProduXion Manager® from Aries Systems Corporation

**First Author:**	Shengyong Xu
**First Author Secondary Information:**	
**Order of Authors:**	Shengyong Xu
	Shijun Xiao
	Shilin Zhu
	Xiaofei Zeng
	jing luo
	Jiaqi Liu
	tianxiang gao
	Nansheng Chen
**Order of Authors Secondary Information:**	
**Response to Reviewers:**	Dear Editors:<br><br>We are submitting the revised manuscript entitled “A draft genome assembly of the Chinese sillago (Sillago sinica), the first reference genome for Sillaginidae fishes”. Attached please find our detailed responses to specific points raised by both reviewers. All authors greatly appreciate the feedback and we have used the suggestions to guide our revisions. We have made detailed revisions to our manuscript and have clearly answered all questions posed by the editor and the reviewers.<br><br>We believe the quality and clarity of the manuscript has improved greatly due to the feedback of the reviewers. We hope that the paper is now in a form suitable for publication in Giga Science as a Data Note.<br><br>Yours sincerely,<br><br>Prof. Dr. Tianxiang Gao<br>Fishery College of Zhejiang Ocean University<br>Zhoushan, Zhejiang, China<br>Email: gaotianxiang0611@163.com<br>March 27, 2018<br><br><br><br>Comments from the editor:<br>Their reports are below. Please also take a moment to check our website athttps://giga.editorialmanager.com/ to download an annotated version of the manuscript kindly provided by reviewer 1, that was saved as attachment.<br>Reply: We have downloaded the comments from the reviewer1 and used it to revise the manuscript according to all the suggestions.<br><br>In the revised manuscript, please also include the fishbase ID for the species. Before submitting your revised manuscript, please make sure all raw data is submitted to the SRA, with all relevant accession numbers cited in the data availability section of the paper.<br>Reply: We have included the fishbase ID for Sillago sinica in our manuscript. All raw data were also submit to NCBI SRA database, and the accession number was cited in this revised manuscript. (line 283-285)<br><br>Comments from the reviewers:<br>Reviewer reports:<br>Reviewer #1: The authors have produced a high quality, professional genome assembly, using PacBio and Illumina technology. The unexpected high heterozygosity of this fish makes genome assembly difficult with short reads. Use of PacBio long reads in depth produced a high quality genome, even with the awkwardness of the high heterozygosity. Minor corrections to the English and comments on parts of the manuscript are attached as a track changes word document. The annotation process is

somewhat unusual, but the measure of its success lies solely with the % of full length peptides predicted. The CEGMA/BUSCO % of full length genes needs to be added, as the authors only mention the % identified genes. This is an important and critical result for gauging the assembly quality.
Reply: We appreciate the reviewer's comments. Indeed, the high heterozygosity is one of the biggest challenge for many fish genome assembly projects. Our work, an application of PacBio sequencing to assembly fish genome with heterozygosity, could provide valuable reference for other fish genome assembly projects in the research community. We have carefully revised the manuscript according to the reviewers' suggestions for English, annotation, genome assembly evaluation, and all corrections were highlighted by red.

Below are several questions in the track changes from the reviewer 1:
how does the mapping ratio correlate with the heterozygosity measurement? Pretty well by the look.
Reply: Heterozygosity of the genome might influence the paired-end mapping ratio; however, it should be the main factor dominating the read mapping ratio. The completeness and accuracy of genome assembly are two main reasons dominating read mapping ratio.

Was Rfam searched using Infernal? This needs to be put explicitly as it could be interpreted as search by sequence similarity
Reply: This is an embarrassing error. The Rfam annotation were searched using Infernal. We corrected the method description and the revised context were highlighted in red (line 236).

Reviewer #2:
The authors report on the genome assembly of a heterozygous specimen of Sillago sinica, a species of the smelt-whiting family. This species occurs in the northwest Pacific Ocean, and was described in 2011 based on phenotypic and molecular data (COI). Sillaginidae contains several species that are difficult to distinguish morphologically. The authors use an established combination of various sequencing technologies (Illumina HiSeq and long-range PacBio) to generate several assemblies that are subsequently merged. Annotation is performed in silico based on sequence homology with other teleosts and species-specific RNA data. The authors use various methods to assess the quality of their genome assembly.

The paper is a description of the assembly and no novel biological insights are reported. Teleost genomes are notoriously difficult to assemble, often yielding highly fragmented genomes. The combined approach used here delivers a contig continuity that is in the upper range of those reported for fish, and is a clear demonstration of the value of including long-range PacBio for genome assembly. The authors claim that their contig length is remarkably larger than other teleost assemblies, but leave reasons for this (whether of technical or biological nature) unexplored. Nevertheless, this assembly remains fragmented, unordered and lacks chromosome level resolution. The concept of quality as emphasized twice in the title, is therefore ambiguous and this assembly represents a draft genome. That status should be reflected in the title. The authors should address their use of ambiguous quality statements (only x number of contigs, highly contiguous, high quality, remarkably and so forth) throughput the paper. The assembly will be of value for researchers working on Sillaginidae.
Reply: We are grateful to the reviewer for all the constructive suggestions, which help to improve the manuscript greatly. Indeed the genome assembly for the Sillago sinica remains fragmented, and we have re-titled the manuscript as "A draft genome assembly of the Chinese sillago (Sillago sinica), the first reference genome for Sillaginidae fishes" to better reflect the genome quality. For the reviewer's concern regarding to genome quality evaluation, a separated section (Genome quality evaluation) was described to assess the assembly quality (line 168). To clarify the quality of genome assembly, we have revised the context of the assembly throughout the manuscript according to the reviewer's comments. All correction were highlighted in red (title and line 267).

Figure and table legends are not complete and absent in most cases. It should be possible to understand figures and tables without the main text.
Reply: This is embarrassing that we neglected figure and table legends. In present

Powered by Editorial Manager® and ProduXion Manager® from Aries Systems Corporation

version, we have completed the legends for figures and tables to facilitate the reader to understand the contents. The revision were shown in red (line 310-312, 314-318, 312-313, 329-330, 332-334).

Minor:
Numerous grammatical and or spelling errors appear throughout the text.
Lines 127-128:" After removing adaptor sequences, we obtained 3.4 million subreads (totally 27.2 Gb) with a contig N50 length of 12.96kb (SI Table 3, SI Figure 3)." A read is not a contig.
Reply: We corrected the contig N50 length to read N50 length (line 148).

In my opinion, for this report it is irrelevant that this project contributes to the future Genome 10K project. If this project was funded through Genome 10K that can be mentioned elsewhere.
Reply: This work was not funded by Genome 10K project, but the data and assembly results could be useful for other genome projects, such as Genome 10K, and for genomic analysis for Sillaginidae. We have deleted the description related to the Genome 10K project in the manuscript.

Reviewer #3: The purpose of the study, High-quality genome assembly of the Chinese sillago, by Xu et al., was to assemble and annotate a long-contiguity genome of the Chinese sillago, Sillago sinica.

High molecular weight genomic DNA with fragment size around 20kb was extracted from muscle tissues. Five paired-end (PE) Illumina libraries with insert sizes 250 bp, 300 bp, 500 bp, 800 bp, and 2kb were sequenced on the Illumina HiSeq platform. The genome size was estimated using a k-mer approach. After constructing a pilot genome assembly, two additional genomic libraries with insert size 20 kb were prepared and sequenced using five SMRT cells on PacBio Sequel. The PacBio sequences were assembled, first by using FALCON and second by using Canu genome assembler. Both assemblies were merged using Genome Puzzle Master (GPM) and then the integrated assembly was polished using Illumina data to generate a final, integrated and polished, genome assembly of S. sinica. The genome quality evaluation was performed using CEGMA and BUSCO to validate the completeness of eukaryotic ortholog genes in the genome assembly.

Based on the distribution of k-mer of size 17, the genome size was estimated to be 543 Mb with 66x estimated coverage, 0.76% heterozygosity (which is higher than that of other fish species), and 12.7% repeat content. The genome assembly based only on Illumina data was of low quality with a total size of 624Mb and 3.2Kb contig N50. The Falcon/Canu hybrid genome assembly based on PacBio sequences (and polished with Illumina sequences) was 534Mb in size with a 2.6Mb contig N50, 802 contigs, and 96% complete orthologous genes. A total of 22,122 protein coding genes were annotated.

The manuscript by Xu, et al., reports a high-quality genome assembly of the Chinese sillago, the first among species within Sillaginidae. The data is solid with appropriate analyses and appropriate interpretations of the results. However, minor revisions are required.

1. Lines 61-62: Authors' claim "Owing to similar phenotypic characteristics, delineation and identification of Sillaginidae species often confuse the taxonomists" is not supported by reference.
Reply: We have added references for the sentence (line 61). Owing to similar phenotypic characteristics, many misidentified species were identified in the last decade using molecular markers. Therefore, we believe that genomic data in this work could prompt the species identification studies for the Sillaginidae in the future.

2. Lines 62-64: Authors' claim "Rapid environment changes resulted from anthropogenic activities can force Sillaginidae species adapt to diversifying situations, leading to further diversification and speciation" is not supported by reference.Lines 65-66: Authors' statement "Numerous cryptic lineages were identified in S. sihama complex by using phenotypic traits and molecular markers in the Northwestern Pacific" does not have reference to support it.

Powered by Editorial Manager® and ProduXion Manager® from Aries Systems Corporation

	Reply: A reference was added to support the sentence. (line 64)<br><br>3.Lines 66-67: Authors stated, "five recently identified Sillago species were misidentified as S. sihama", but did not provide information on whether the method used for identification was solely phenotypic or both phenotypic or molecular. Lines 70-71. Authors stated, "among Sillaginidae species, the Chinese sillago Sillago sinica is one the most recently identified Sillaginidae species in the Northwestern Pacific", but did not provide information on the previously used method to identify S. sinica.<br>Reply: We appreciate the suggestions. Previous misidentification were made solely on phenotypic data. However, the application of molecular markers helped to reveal cryptic species among Sillaginidae species in recent years. We have added the methods and information used to the species identification. The revision were highlighted in red (69-71).<br><br>5. Line 72. Authors stated, "Due to their phenotypic similarity, S. sinica was previously misidentified as S. sihama", but did not support the statement by reference.<br>Reply: References were added for the sentence (line 74).<br><br>6. Lines 73-74. Authors stated, "two species are different because S. sinica inhabits cold-temperate environment while S. sihama inhabits warm-temperate environment", but did not provide any reference to support that the same species can't inhabit different environments.<br>Reply: A reference was added to support the sentence (line 75).<br><br>7. Lines 74-77. Authors concluded, "It is thus essential to sequence the genome of S. sinica", and claimed that sequencing the genome of S. sinica "will improve taxonomy, and may help to reveal insights into evolutionary history of Sillaginidae species and the role of environment changes in rapid genetic diversification and speciation". However, authors did not provide any support for their claims.<br>Reply: References were added to support the context. Previous studies have shown the contribution of the genome on environmental adaptation and evolutionary studies, we also cited the related researches for other fish species. (line 78)<br><br>8. Line 87. Authors stated, "we collected fresh muscle tissue", however it is not clear what type of muscle tissue was collected (red muscle tissue or white muscle tissue?).<br>Reply: Epaxial white muscle tissues were collected for DNA extraction and sequencing. We have added the information into the manuscript and highlighted the revision in red (line 88).<br><br>9. Lines 91-92. Authors claimed, "a main band around 20 kb indicating high-quality for PacBio Sequel platform", but did not provide reference to support why 20 kb indicates quality. Perhaps they mean the DNA is high molecular weight, or is long enough for sequencing on the PacBio platform.<br>Reply: The resolution of the conventional agarose gel electrophoresis was 200 bp to 20 kb. In this work, 20 kb libraries were prepared for PacBio sequencing; therefore, a single band above 20 kb on an agarose gel indicated that lengths of DNA in the band were at least 20kb, and the integrity of DNA molecules satisfied the requirement for PacBio library construction (https://www.pacb.com/wp-content/uploads/2015/09/Guide-Pacific-Biosciences-Template-Preparation-and-Sequencing.pdf). (line 93)<br><br>10. Lines 95. Authors stated, "we also sequenced the genomic DNA using Illumina DNA sequencing technologies", but did not provide information on the amount of DNA used for sequencing.<br>Reply: 20 ug DNA molecules were used for library construction and Illumina DNA sequencing. We have added the information in the context (line 97-98).<br><br>Lines 96-97. Authors stated, "Five paired-end libraries were constructed with insert sizes of 250 base pairs (bp), 300 bp, 500 bp, 800 bp, 2 kb and generated a total of ~42 Gb sequence data", but did not provide the reason(s) of using different insert sizes, the technical type of libraries (was the 2 kb library a mate-pair library, or a paired-end library?), and the meaning of ~42 Gb sequence data - what coverage of the genome was generated by each library?.<br>Reply: We thanks reviewer for the important concern. Sequencing with multiply libraries for various insertion length was a traditional strategy for genome assembly

	using Illumina sequencing platform. Before the application of PacBio platform, we first tried to assess the feasibility to assembly the Chinese sillago solely using the Illumina platform, therefore, various insertion length (250 base pairs (bp), 300 bp, 500 bp, 800 bp, 2 kb) were generated. The library of 2kb was a mate-pair library. We have corrected the library type of 2kb (line 97).<br>We are sorry that we made a mistake to calculate the sequencing amount (~42Gb) for 300 bp library as the total NGS sequencing data. Actually, 35, 42, 31, 39 and 18 Gb data were generated, representing the genome coverage of 67X, 81X, 60X, 75X and 35X, for 250 bp, 300 bp, 500 bp, 800 bp, 2 kb, respectively, resulting into a total of 165 Gb NGS data (a coverage of ~317X). We added the information into the revised manuscript (line 98-99) and Table 1.<br><br>11. Line 99. Authors stated, "Raw reads were analyzed using FastQC and then filtered using HTQC". However, the following things are not clear: a) the kind of analysis performed by using FastQC, and b) the type of sequence filtered using HTQC.<br>Reply: We thank the reviewer for this reminding. FastQC was used for quality control, including the base and read quality evaluation, and HTQC was used for quality and length trimming. We have added the information in the revised manuscript (line 101-102).<br><br>12. Lines 100-105. Authors stated, "reads were filtered in the following filtering steps: 1) Removing adaptors…………..; 2) Removing read pairs……….;3) Trimming ambiguous or low quality fragments………..; 4) Removing read pairs………", but it is not clear which software performed which step.<br>Reply: We thank reviewer for the reminding. All above analysis were performed with FastQC and HTQC. We have added the information in the manuscript (line 103).<br><br>13. Line 105. What does "A single peak around 45%"mean? Also, which software was used to see GC distribution?<br>Reply: GC distribution in reads were calculated and analyzed by FastQC. We have added the information into the manuscript (line 107-108).<br><br>14. Line 106-107. Authors stated, "After searching against to non-redundant nucleotide (nt) database with BLASTN, we found that the best hits were enriched to closely related fish species". However, authors could provide the following information: the type of sequence that was searched against non-redundant nucleotide database using BLASTN, the reason of the search, and the place where the result of this analysis could be found.<br>Reply: Short reads from NGS might contained the contamination during library construction and sequencing. To check if there is any obvious contamination in the sequencing data, we randomly selected 10,000 paired sequencing reads and searched against the NT database. From our result, no obvious contamination was observed. We have revised the manuscript (line 113-114) and added the table in the revised Supplementary Information Table 2.<br><br>15. Line 108-109. What are the common names of the "closely related fish species"?<br>Reply: We have added the common names of the closely related fish species (line 111-113).<br><br>16. Line 110. Authors stated, "We estimated the genome size of the Chinese sillago by analyzing the 17-mer depth distribution". However, it is not clear what type of reads were used for creating 17-mer distribution (raw reads or cleaned sequence reads and from which molecular libraries). Further, authors conducted K-mer based method of genome size estimation. However, there is no information on the following things: a) Why was 17-mer chosen? b) Was the process iterated for different k-mers? How was k-mer distribution generated? How was the peak position determined? Which software was used for genome size estimation based on 17-kmer? The author use an ambiguous citation (citation #16) to "gce" with no further information.<br>Reply: The comments from the reviewer was very important. Clean reads after quality trim were used for all sequencing analysis, including Kmer analysis. Previous studies1 often require the Kmer space to be at least 5 times larger than the genome size (4K>5*G), and the larger the better. Kmer size of 17 satisfy the requirement.<br>17-mer were generated using gce software1, which called jellyfish for K-mer distribution generation. The peak position was also determined by gce. We have added

the reference in the revised manuscript (line 121). In addition, we updated the reference for the Kmer method for genome size estimation (line 116). We applied 17, 21 and 27 for Kmer size, and found that the estimated genome size ranged from 519 to 524 Mb. The resulted were added to the Supplementary Information (SI Table 3) and the information was added to the revised manuscript (line 123-125).

17. Line 113. Authors provided the meaning of N17-mer and D17-mer explicitly but not that of 'G'.
Reply: The G here mean the estimated genome size. We have added the information in the revised manuscript. (line 120)

18. Lines 114-115. Authors stated, "For our data, N17-mer was 37,811,957,476 and D17-mer was 66, suggesting an estimated genome size of 524 Mb", but did not state which value suggests the estimated genome coverage.
Reply: Thanks for reviewer's comments. From the K-mer method, the peak of K-mer distribution was the estimation of the genomic sequencing coverage, which was 66 in this work (line 121-122).

19. Lines 115-116. How were "heterozygosity" and "repeat content" estimated?
Reply: Thanks for reviewer's comments. The heterozygosity and repeat content were estimated from the statistical models from the Kmer distribution. The underlying principle were comprehensively explained in the article of gce software1. We have added the information in the revised manuscript (line 126 and 127).

20. Line 120. Authors should point out what "artificial breeding" does to the genome heterozygosity.
Reply: Thanks a lot for the reviewer's comment. Many artificial breeding techniques in aquaculture, such as inbreeding and gynogenesis, could effectively minimize the genomic heterozygosity and potentially reduce the difficulty of the genome assembly. We have added reference for the genome assembly of a farmed fish species, grass carp2, in the revised manuscript (line 131-133).

21. Line 120. What were the reason for generating a "pilot assembly"?
Reply: We thank the reviewer for the important concern. As our response to the above comment, we first tried to assembly the Chinese sillago solely using the Illumina platform with various insertion length libraries, which was called the pilot assembly. The assembly from the pilot assembly was high fragmented, we therefore instead applied the PacBio platform for the genome assembly. We have revised the manuscript to clarify (line 134-145).

22. Line 121. Authors used "Platanus package" to generate "pilot genome assembly". However, it is unknow why Platanus package was used and how Platanus package was used. What parameters were employed and was another assembler used as well for comparison?
Reply: We appreciate this important concern. Genomic heterozygosity is one of the biggest challenge of many complex genome assembly.3 Platanus package were designed for heterozygous genome assembly and exhibited excellent performance in several complex genome3. Therefore, we used the package for our pilot genome assembly. The default parameters were used for the genome assembly. We did not applied other assembler for the genome since we focused on PacBio assembly in this work (line 134-139).

23. Line 122-123. Authors stated, "genome assembly was of low-quality partly due to its high genomics heterozygosity", however it is unknown why the pilot genome assembly was considered of low quality. Also, assuming by "low-quality" the authors are referring to contig N50, a low value here is much more likely to be attributed to the insert sizes of the molecular library (maximum size 2 kb) as opposed to heterozygosity.
Reply: The comments helped us to improve the context from line 137 to 142. The assembly solely using NGS data was highly fragmented and the low-quality here was referred to the continuity, namely contig N50. We have revised the manuscript and deleted "low-quality" and its ambiguous correlation to"high genomics heterozygosity".

24. Line 125-126. Authors stated, "We prepared two 20 kb genomic DNA libraries, which we sequenced using PacBio Sequel using five SMRT cells, generating 27.3 Gb

	raw reads", but the reason of using long read sequences to assemble the genome is not mentioned. Again, the meaning of 27.3 Gb raw DNA reads is unknown, what coverage was generated?<br>Reply: Because of short reads, the assembly using traditional NGS short sequencing data resulted into highly fragmented genome for Chinese sillago in this work. Previous studies illuminate the excellent performance of PacBio long reads on complex genome assembly4,5; we therefore applied PacBio to generate long reads, aiming to generate longer contig assembly for the genome. The PacBio sequencing data of 27.3 Gb represented the ~53X coverage of the genome. We have added the information in the revised manuscript (line 148).<br><br>25. SI Figure 2. On graph authors could indicate the portions of the graph that show error k-mers, true k-mers, peak by heterozygotic alleles, main peak, and repeats.<br>Reply: We have revised SI Figure 2 to indicate the error k-mers, true k-mers, peak by heterozygotic alleles, main peak, and repeats.<br><br>26. SI Figure 4. Genome sequence validation using NGS reads from the libraries with various length was performed and the result is shown in SI Figure 4 but nothing is presented on text about it.<br>Reply: We have clarified the context of SI Figure 4 in the line 179-181 in the revised manuscript, as a supporting evidence of genome quality.<br><br>27. The text needs to be copy-edited to fix a number of simple English grammatical errors.<br>Reply: We have carefully revised the English grammatical errors through the manuscript. All corrections were highlighted in red.<br><br>References<br>1Liu, B. et al. Estimation of genomic characteristics by analyzing k-mer frequency in de novo genome projects. Quantitative Biology 35, 62-67 (2013).<br>2Wang, Y. et al. The draft genome of the grass carp (Ctenopharyngodon idellus) provides insights into its evolution and vegetarian adaptation. Nature Genetics 47, 625-631 (2015).<br>3Kajitani, R. et al. Efficient de novo assembly of highly heterozygous genomes from whole-genome shotgun short reads. Genome Research 24, 1384-1395 (2014).<br>4Conte, M. A., Gammerdinger, W. J., Bartie, K. L., Penman, D. J. & Kocher, T. D. A high quality assembly of the Nile Tilapia (Oreochromis niloticus) genome reveals the structure of two sex determination regions. Bmc Genomics 18, 341 (2017).<br>5Fu, X. et al. Long-read sequence assembly of the firefly Pyrocoelia pectoralis genome. Gigascience 6, 1-7 (2017).
**Additional Information:**	
**Question**	**Response**
Are you submitting this manuscript to a special series or article collection?	No
**Experimental design and statistics**<br><br>Full details of the experimental design and statistical methods used should be given in the Methods section, as detailed in our Minimum Standards Reporting Checklist. Information essential to interpreting the data presented should be made available in the figure legends.<br><br>Have you included all the information requested in your manuscript?	Yes

**Resources**<br><br>A description of all resources used, including antibodies, cell lines, animals and software tools, with enough information to allow them to be uniquely identified, should be included in the Methods section. Authors are strongly encouraged to cite Research Resource Identifiers (RRIDs) for antibodies, model organisms and tools, where possible.<br><br>Have you included the information requested as detailed in our Minimum Standards Reporting Checklist?	Yes
**Availability of data and materials**<br><br>All datasets and code on which the conclusions of the paper rely must be either included in your submission or deposited in publicly available repositories (where available and ethically appropriate), referencing such data using a unique identifier in the references and in the “Availability of Data and Materials” section of your manuscript.<br><br>Have you have met the above requirement as detailed in our Minimum Standards Reporting Checklist?	Yes

Powered by Editorial Manager® and ProduXion Manager® from Aries Systems Corporation

Manuscript
Click here to access/download;Manuscript;manuscript_Sillago_sinica_21_8.
Click here to view linked References

A draft genome assembly of the Chinese sillago (*Sillago sinica*), the first reference genome for Sillaginidae fishes

Shengyong Xu[1]*, Shijun Xiao[2]*, Shilin Zhu[2], Xiaofei Zeng[2], Jing Luo[3], Jiaqi Liu[2], Tianxiang Gao[1,#], Nansheng Chen[4,5,#]

[1] Fishery College, Zhejiang Ocean University, Zhoushan, Zhejiang, China

[2]Wuhan Frasergen Bioinformatics Co., Ltd., Wuhan, Hubei, China

[3]School of Life Sciences, Yunnan University,Kunming, Yunnan,China

[4]Institute of Oceanology, Chinese Academy of Sciences, Qingdao, Shandong, China

[5]Department of Molecular Biology and Biochemistry, Simon Fraser University, Burnaby, Canada

Abstract

Background

Sillaginidae, also known as smelt-whitings, is a family of benthic coastal marine fishes in the Indo-West Pacific that have high ecological and economic importance. Many Sillaginidae species, including the Chinese sillago (*Sillago sinica*) have been recently described in China, providing valuable material to analyze genetic diversification of the family Sillaginidae. Here, we constructed a reference genome for the Chinese sillago, with the aim to setup a platform for comparative analysis of all species in this family.

Findings

Using the single-molecule real-time DNA sequencing platform PacBio Sequel, we generated ~27.3 Gb genomic DNA sequences for the Chinese sillago. We reconstructed a genome assembly of 534 Mb using a strategy that takes advantage of complementary strengths of two genome assembly programs, Canu and FALCON. The genome size was consistent with the estimated genome size based on *K*mer analysis. The assembled genome consisted of 802 contigs with a contig N50 length of 2.6 Mb. We annotated 22,122 protein-coding genes in the Chinese sillago genomes using a *de novo* method as well as RNA-seq data and homologies to other teleosts. According to the phylogenetic analysis using protein-coding genes, the Chinese sillago is closely related to *Larimichthys Crocea* and *Dicentrarchus labrax* and diverged from their ancestor around 69.5 - 82.6 million years ago.

Conclusions

Using long reads generated with PacBio sequencing technology, we have built a draft genome assembly for the Chinese sillago, which is the first reference genome for Sillaginidae species. This genome assembly sets a stage for comparative analysis of the diversification and adaptation of fishes in Sillaginidae.

Key Words: Sillaginidae, Chinese sillago, PacBio sequencing, Canu, FALCON, genetic diversification

Data description

The fish family Sillaginidae consists of demersal marine fishes commonly known as sand whitings or sand borers[1] that inhabit inshore waters throughout the Indo-West Pacific[2,3]. As ecologically and commercially important marine organisms, Sillaginidae species play important roles in the commercial fisheries of Pakistan, Australia, China, Malaysia, Thailand and Philippines[1,4]. Owing to similar phenotypic characteristics, delineation and identification of Sillaginidae species often confuse the taxonomists.[5,6] Additionally, rapid environment changes resulting from anthropogenic activities can force Sillaginidae species to adapt to diversifying situations, leading to further diversification and speciation.[7,8] Numerous cryptic lineages were identified in *S. sihama* complex by using phenotypic traits and molecular markers in the Northwestern Pacific.[9] For example, five recently identified Sillago species were misidentified as *S. sihama* solely using phenotypic data[5,6,10-12]. Therefore, it is essential to investigate Sillaginidae species at the genetics level to identify molecular features for accurate characterization of different species, and for understanding rapid genetic diversification and speciation. Using a combined method with morphological and phylogenetic analysis of the mitochondrial DNA cytochrome oxidase subunit I (COI) gene, the Chinese sillago, *Sillago sinica,* (Figure 1, Fishbase ID: 65964) is one of the most recently identified Sillaginidae species in the Northwestern Pacific[5]. Due to their phenotypic similarity, *S. sinica* was previously misidentified as *S. sihama*.[5] However, these two fish species are different because *S. sinica* inhabits cold-temperate environment while *S. sihama* inhabits a warm-temperate environment.[9] It is thus essential to sequence the genome of *S. sinica*, which will improve taxonomy, and may help to reveal insights into evolutionary history of Sillaginidae species and the role of environment changes in rapid genetic diversification and speciation.[5,6,13,14]

Here we present a reference genome assembly for *S. sinica* constructed using long reads generated by the PacBio DNA sequencing platform Sequel, and using a genome assembly strategy taking advantage of two genome assemblers Canu[15] and FALCON[16]. This genome assembly of the Chinese sillago is the first genome constructed for the family Sillaginidae. The completeness and continuity of the genome provided valuable genomic resources for studies on evolutionary history of the rapid speciation processes of Sillaginidae species.

Sample and DNA extraction

To obtain enough genomic DNA for PacBio Sequel platform (Pacific Biosciences of California, Menlo Park, CA, USA), we collected fresh epaxial white muscle tissue from a Chinese sillago fish in Zhoushan city, Zhejiang province. The sample was quickly frozen in liquid nitrogen for one hour before storing at −80°C. Genomic DNA was extracted using

standard phenol/chloroform extraction protocol. The integrity of genomic DNA molecules was checked using agarose gel electrophoresis, showing a main band around 20 kb and satisfying the requirement for PacBio library construction by the manufacturer's protocol.

Genome size estimation

To estimate the Chinese sillago genome size, we also sequenced the genomic DNA using Illumina Next-Generation Sequencing (NGS) technologies. Four paired-end libraries with insert sizes of 250 base pairs (bp), 300 bp, 500 bp, 800 bp and one mate-pair 2 kb library were constructed from 20 ug DNA molecules. 35, 42, 31, 39 and 18 Gb data were generated, representing the genome coverage of 67X, 81X, 60X, 75X and 35X, for 250 bp, 300 bp, 500 bp, 800 bp, 2 kb, respectively, resulting in a total of ~ 165.5 Gb NGS data (a coverage of ~317X) (Table 1, SI Table 1) on the Illumina HiSeq X Ten platform (Illumina Inc., San Diego, CA, USA).

The quality of raw reads were evaluated using FastQC (FastQC, RRID:SCR_014583)[17] and then filtered by quality and length using HTQC[18]. Low quality bases and reads were filtered in the following filtering steps with FastQC and HTQC: 1) Removing adaptor sequences introduced during sequencing library construction; 2) Removing read pairs if the average base quality was lower than 20 for any of the two ends; 3) Trimming ambiguous or low quality fragments at two ends of reads within a window size of 5 bp and an average quality threshold of 20; 4) Removing read pairs if any of the two reads had a read length shorter than 75. Using FastQC for cleaned sequencing reads, a single peak around 45% were identified in GC distribution (SI Figure 1). 10,000 reads pairs were randomly selected and were searched against non-redundant nucleotide (nt) database with BLASTN[19]. We found that the best hits of reads were enriched for closely related fish species[20], including medaka (*Oryzias latipes)*, large yellow croaker (*Larimichthys corcea*), common carp (*Cyprinus carpio*) , seabass (*Dicentrarchus labrax*) and zebrafish (*Danio rerio*) (SI Table 2), indicating no obvious contamination was observed in the sequencing data.

By analyzing the 17-mer depth distribution from 300 bp library cleaned sequencing reads in gce software[21], we estimated the genome size of the Chinese sillago using the following equation:

$$G = N_{17\text{-mer}} / D_{17\text{-mer}}$$

Where the $N_{17\text{-mer}}$ was the total number of 17-mers, $D_{17\text{-mer}}$ denoted the peak frequency of 17-mers estimated and G represented the estimated genome size. $N_{17\text{-mer}}$ was 37,811,957,476 in our data, and $D_{17\text{-mer}}$ was estimated as 66 in gce software[21], suggesting the coverage of sequencing data for the Chinese sillago genome was about 66 and an estimated genome size of 524 Mb according to the above equation. We also used the

*K*mer of 21 and 27 for the analysis, and found the estimated genome size ranged from 519 to 524 Mb (SI Table 3). Meanwhile, we observed a heterozygous and a repeat peak (SI Figure 2), with an estimated heterozygosity of 0.66-0.76% (6.6-7.6 SNP / 1000 nt) and a repeat content of 11.3-12.7% for the Chinese sillago, according to the statistical model in gce software[21]. The heterozygosity of our sample was noticeably higher than other fish species in previous genome studies[22-24], partly because the Chinese sillago sample used in this project was collected directly from the wild environment without further artificial inbreeding. Many artificial breeding techniques in aquaculture, such as inbreeding and gynogenesis, could effectively decrease the genomic heterozygosity and potentially reduce the difficulty of the genome assembly.[24]

Using short reads with various insert lengths, we performed a pilot assembly solely with NGS data. Genomic heterozygosity is one of the biggest challenge of many complex genome assembly and the Platanus (Platanus, RRID:SCR_015531) package were designed for heterozygous genome assembly.[25] Therefore, the Platanus package[25] with the default parameters was applied for our pilot genome assembly. As a result, we constructed a 624 Mb genome assembly with more than 1 million contigs and a contig N50 length of 3.2 kb (Table 2).

Genome assembly with long PacBio reads

The pilot assembly with traditional short sequencing data resulted into high fragmented assembly reference genome for Chinese sillago, especially on the continuity of contig level. Previous studies illuminate the excellent performance of PacBio long reads on complex genome assembly.[26,27] We therefore applied PacBio to generate long reads, aiming to generate longer contig assembly for the genome. To this end, we prepared two 20 kb genomic DNA libraries, which were sequenced using PacBio Sequel using five SMRT cells, generating 27.3 Gb raw DNA reads with a genomic coverage of ~53 X (Table 1, SI Table 4). After removing adaptor sequences, we obtained 3.4 million subreads (totally 27.2 Gb) with a read N50 length of 12.96 kb (SI Table 5, SI Figure 3).

Because of the high heterozygosity for the Chinese sillago, we first used FALCON (FALCON, RRID:SCR_016089)[16] for genome assembly. With the parameter of length_cutoff set at 10 kb and pr_length_cutoff at 8 kb, we produced a 546 Mb genome assembly for the Chinese sillago, which agreed well with the estimated genome size in 17-mer analysis (above). The genome assembly consisted of 2,066 contig with a N50 length of 1.5 Mb (Table 2). Meanwhile, we also applied Canu[15] v1.4 (Canu, RRID:SCR_015880) to assemble the genome with the CorrectedErrorRate parameter set at 0.052. As a result, we obtained a second Chinese sillago genome of 527 Mb, with 1,349 contigs and contig N50 of 1.62 Mb (Table 2). Thus, both assemblies have similar genome sizes and excellent continuity. We then used Genome Puzzle Master (GPM)[28] to merge

the two genome assemblies into an integrated genome by tracking the overlapping relationships between contigs of the two genome assemblies, and applied Redundans[29] (v0.13c) to remove the sequence redundancy. The resulting genome assembly was further polished using NGS data, which were used in the genome survey analysis above. The contig N50 length of the final 534 Mb Chinese sillago genome assembly reached 2.6 Mb (Table 2). The contig N50 of the Chinese sillago was much higher than those of previous fish genome assemblies constructed using NGS DNA sequencing technologies, and was comparable with those of recently reported model fish species[30,31]. (Figure 2)

Genome quality evaluation

To validate the completeness of the Chinese sillago genome assembly, we subjected the sequences to CEGMA (CEGMA, RRID:SCR_015055)[32] and BUSCO (BUSCO, RRID:SCR_015008)[33] evaluation. More than 96% of core eukaryotic genes were successfully identified in the Chinese sillago genome in both CEGMA (SI Table 6) and BUSCO (SI Table 7) analyses and more than 92.8% were detected as complete single-copy BUSCO genes, implying a high completeness of the Chinese sillago genome assembly.

To further evaluate the accuracy of the Chinese sillago genome assembly, we aligned the NGS-based short reads from whole-genome sequencing data against the genome assembly using BWA (BWA, RRID:SCR_010910)[34]. We found that 98.4% of the reads were reliably aligned to the genome assembly, and 95.8% of the reads were properly aligned to the genome with their mates. The insertion length distribution for sequencing library of 250 bp, 300 bp, 500 bp, 800 bp, 2 kb exhibited a single peak around the sequencing library length chosen (SI Figure 4), illuminating the high quality of the genome assembly. Using genomic homozygous mutations detected from the NGS data, we estimated the genome accuracy at the base level reached 99.997%.

Repeat annotation

We annotated repetitive elements in the Chinese sillago genome using Tandem Repeat Finder[35]. To identify transposon elements (TE), RepeatModeler (RepeatModeler, RRID:SCR_015027) was used to identify *de novo* repeat types in the genome. The Repbase database[36] of known repeats and a *de novo* repeat library generated by RepeatModeler were used. The TEs in the Chinese sillago genome were then identified by mapping to the library using the software RepeatMasker (RepeatMasker, RRID:SCR_012954)[37].

We found that tandem repeat content in Chinese sillago (4.69%) was much higher than those in *Gasterosteus aculeatus* (2.03%), *Larimichthys corcea* (2.7%), *Oryzias*

latipes (0.92%) and *Dicentrarchus labrax* (2.8%). However, the content of TEs (12.86%) of the Chinese sillago was lower than those of the above fish species (SI Figure 5, SI Table 8), leading to an overall lower content of repetitive sequences in the Chinese sillago genome, which might be a reason for the relatively small genome size of Chinese sillago.

RNA preparation and sequencing

We also sequenced, using Illumina sequencing technologies, cDNA libraries prepared from the same Chinese sillago fish individual used for genome annotation. Tissues of ocular, skin, muscle, gonadal, intestinal, liver, kidney, blood, gall and air bladder tissues were collected and RNAs were extracted with TRIZOL Reagent (Invitrogen, USA). RNAs were then balanced mixed for the sequencing. The absorbance of 1.90 at 260 nm/280 nm and the RIN of 9.1 were obtained for the purified RNA sample by Nanodrop ND-1000 spectrophotometer (LabTech, USA) and 2100 Bioanalyzer (Agilent Technologies, USA), respectively.

According to the protocol suggested by the manufacturer, one microgram of RNA was reverse transcribed using Clontech SMARTer cDNA synthesis kit, and was further fragmented using divalent cations for NGS sequencing. The paired-end library was prepared following the manual of the Paired-End Sample Preparation Kit (Illumina Inc., San Diego, CA, USA). Finally, the library with an insert length of 300 bp was sequenced by Illumina HiSeq X Ten in 150PE mode (Illumina Inc., San Diego, CA, USA). As a result, we obtained ~10.4 Gb transcriptome data from RNA-seq (Table 1, SI Table 1).

Gene and functional annotation

To annotate genes in the Chinese sillago genome, gene prediction was performed with *de novo*, homology-based and transcriptome sequencing-based method. We first used Augustus (Augustus: Gene Prediction, RRID:SCR_008417)[38] to predict protein-coding genes in the Chinese sillgo genome. Then, protein sequences of closely related fish species, including *Danio rerio, Dicentrarchus labrax, Gasterosteus aculeatus, Larimichthys corcea, Oryzias latipes, Takifugu rubripes and Gadus morhua*, were downloaded from Ensembl[20] and aligned against to the Chinese sillago genome using TBLASTN software[39]. GeneWise (GeneWise, RRID:SCR_015054)[40] was then used to define gene models. We also used NGS transcriptome short reads aligned upon the Chinese sillago genome using the TopHat (TopHat, RRID:SCR_013035) package[41], and the gene structures were predicted using Cufflinks (Cufflinks, RRID:SCR_014597)[42]. All gene models were then integrated using MAKER[43] to obtain a consensus gene set (SI Figure 6). Altogether, we annotated 22,122 protein-coding genes in the Chinese sillago genome. The gene number, gene length distribution, CDS length distribution, exon length distribution and intron length distribution were all comparable with those in other teleost

fish species (SI Figure 7, SI Table 9).

To obtain functional annotation of the protein-coding genes in the Chinese sillago genome, we searched the NCBI non-redundant protein (nr), non-redundant nucleotide (nt), and Swissprot database using local BLASTX and BLASTN programs with an e-value threshold of 1e-5[19]. We then searched the Gene ontology (GO)[44] and Kyoto Encyclopedia of Genes and Genomes (KEGG)[45] pathway databases using the software Blast2GO (Blast2GO, RRID:SCR_005828)[46]. As a result, most (21,768) of the 22,122 genes were annotated by at least one database, representing 98.4% of the total genes (SI Figure 8, SI Table 10). We also annotated four types of non-coding RNAs (microRNAs, transfer RNAs, ribosomal RNAs, and small nuclear RNAs) using tRNAscan-SE (tRNAscan-SE, RRID:SCR_010835)[47] and the Rfam database[48] using Infernal (Infernal, RRID:SCR_011809)[49] (SI Table 11).

Gene family identification

In order to identify gene families among fish species in this work, proteins of the longest transcripts of each individual genes from the Chinese sillago and other fish species, including *Dicentrarchus labrax, Larimichthys corcea, Astyanax mexicanus, Danio rerio, Gadus morhua, Gasterosteus aculeatus, Lepisosteus oculatus, Oryzias latipes, Takifugu rubripes, Xiphophorus maculates* and *Callorhynchus milii,* were aligned to each other with BLASTP[19] programs with an e-value threshold of 1e-5. The HSP segments were concatenated by Solar, and H-scores were calculated from Bit-score. At last, gene families were obtained by clustering of homologous gene sequences using H-scores in Hcluster_sg software. As a result, 15,022 gene families were constructed for the Chinese sillago (Figure 3).

Phylogenetic analysis for Chinese sillago and fishes with public genome

To generate the phylogenetic relationship of Chinese sillago with other fish species, the coding sequences of single-copy gene families among all species were extracted and aligned with the guidance of protein alignment from ClustalW program[50] and the alignment were concatenated as a single data set. The maximum-likelihood method implemented in the PhyML (PhyML, RRID:SCR_014629) package[51] with the JTT+G+F model were used to construct the phylogenetic tree from the super-alignment of the coding sequences. The MCMCtree program in the PAML (PAML, RRID:SCR_014932) package was used to determine divergence times with the approximate likelihood method[52] and a molecular clock data from the divergence time between zebrafish and medaka from the TimeTree database[53]. According to the phylogenetic analysis, Chinese sillago were clustered together with *Larimichthys crocea* and *Dicentrarchus labrax,* which was consistent with the fish species taxonomy. Chinese sillago diverged from the common ancestor with

Larimichthys crocea and *Dicentrarchus labrax* around 69.5 - 82.6 million years ago. (Figure 4)

Conclusion

Using long reads from the third-generation PacBio Sequel sequencing platform, we successfully assembled the genome of the Chinese sillago, which represents the first reference genome of all species in Sillaginidae species. The 534 Mb Chinese sillago genome assembly consists of 802 contigs with contig N50 length of 2.6 Mb. The contig N50 is longer than those of most fish genome assemblies, and is comparable with those of recently reported model fish species. The genome base-level accuracy reached 99.997%. We annotated 22,122 protein-coding genes in the Chinese sillago genome assembly. We found that Chinese sillago diverged from the common ancestor of *Larimichthys Crocea* and *Dicentrarchus labrax* around 69.5 - 82.6 million years ago. The genome assembly, together with gene annotation and transcriptome data generated in this work, provided a valuable resource for research on the phylogenetic and adaption investigation of Sillaginidae family.

Ethics Statement

This study was approved by the Animal Care and Use committee of Fishery College of Zhejiang Ocean University.

Availability of supporting data

Raw sequencing data are deposited in the SRA with the accession number of SRR6965224 - SRR6965233. Supporting data and materials, also including the genome assembly and annotations, are available in the *GigaScience* GigaDB database [54].

Competing interests

The authors declare that they have no competing interests.

Funding

This study was supported by a grant from the National Natural Science Foundation of China (No.41776171; No.31572227; No.31602207), Scientific Startup Foundation of Zhejiang Ocean University (No.Q1505) and the Open Foundation from Fishery Sciences in the First-Class Subjects of Zhejiang (No.20160001).

Author Contributions

TXG and NSC conceived the project. SYX collected the samples and extracted the genomic DNA. SJX, SLZ, XFZ and JQL performed the genome assembly and data analysis. TXG, NSC, SJX and JL wrote the paper.

Figure Legends

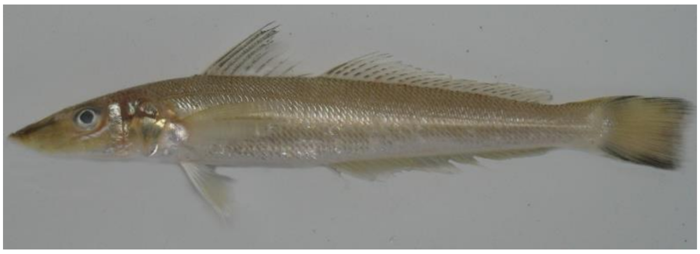

Figure 1. A representative individual of the Chinese sillago.

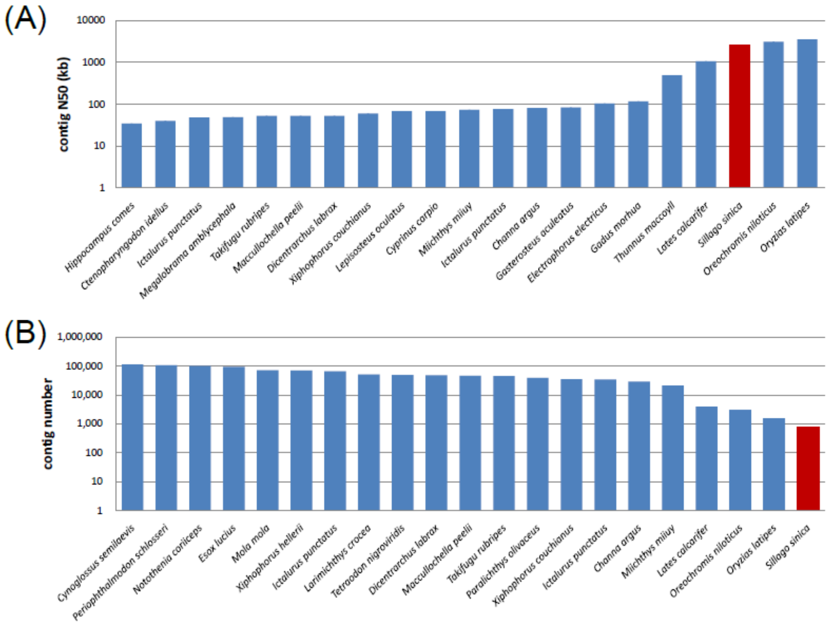

Figure 2. Comparing genome assemblies between Chinese sillago and other fish species. Y-axis represented the contig N50 (A) and contig number (B). Only top 20 public genomes were showed (X-axis) ordered by contig N50 lengths (A) and contig numbers (B).

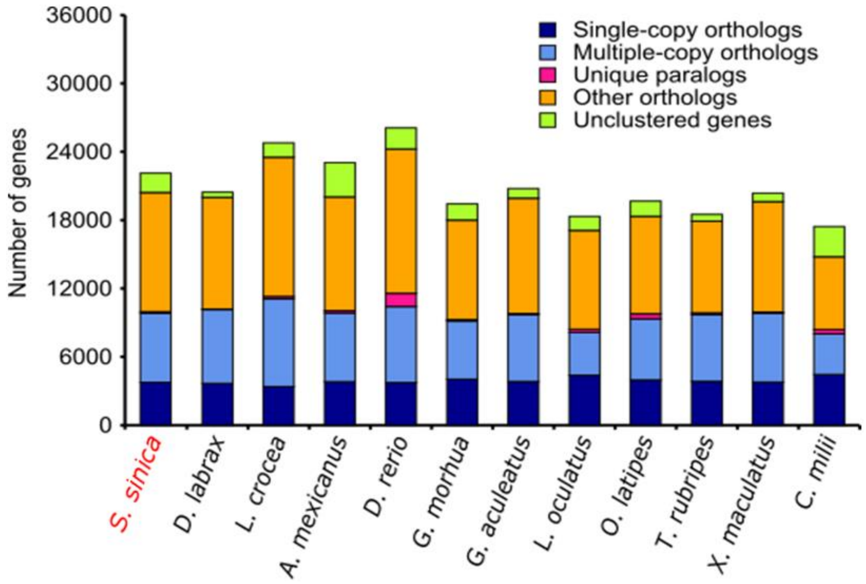

Figure 3. Gene family comparison between Chinese sillago and other fish species (X-axis). The Y-axis represented the gene number for each classes: single-copy (one gene for each species), multiple-copy (more than one gene for each species), unique paralogs (no genes in other species), other orthologs (other cases in gene clusters) and unclustered genes (genes that did not clustered with other genes).

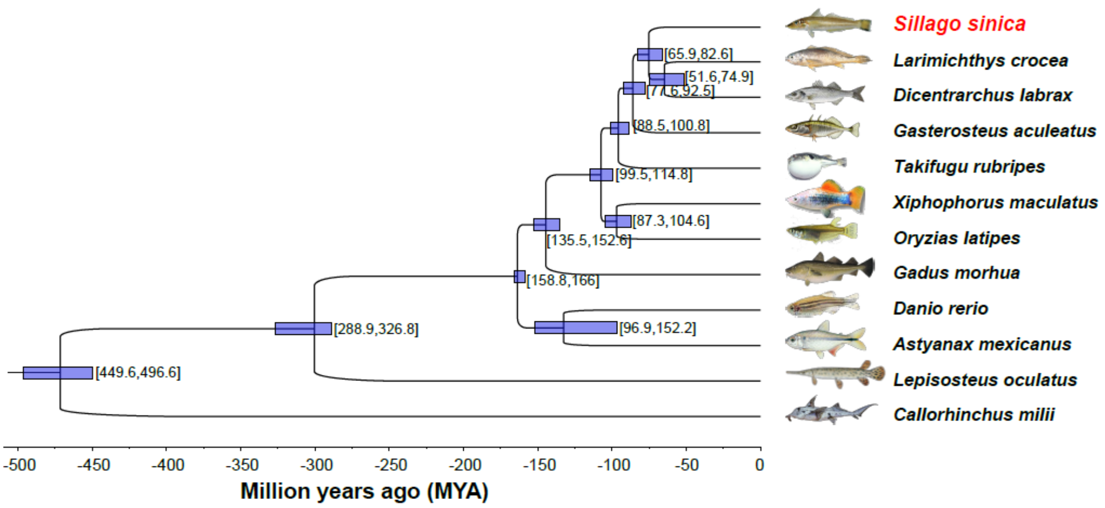

Figure 4. The phylogenetic relationship of Chinese sillago with other fish. The estimated divergence time (Million Years Ago, MYA) were showed in the below coordinates. The blue bars at each branch represented the 95% confidence interval of the species divergence time.

Tables

Table 1 Summary of sequence data from *Sillago sinica*. The sequencing data used in this work. Note that read N50 length for PacBio Sequel were measured for subreads.

Type	Method	Library size (bp)	Data size (Gb)	Read N50 (bp)
**DNA**	HiSeq X Ten	250	34.8	150
		300	42.2	
		500	31.4	
		800	38.8	
		2,000	18.3	
**DNA**	PacBio Sequel	20,000	27.3	12,957
**RNA**	HiSeq X Ten	250	10.5	150

Table 2 Genome assembly statistics for *Sillago sinica.* The assembly result using various method and sequencing data. Note that Platanus were used for NGS data assembly , and FALCON and Canu were used for PacBio data.

Method	Type	Genome size (Mb)	Longest sequence (Mb)	Sequence number	Sequence N50 (Mb)
**Platanus**	contig	624	0.091	1,045,226	0.0032
	scaffold	518	0.735	187,308	0.042
**FALCON**	contig	546	7.8	2,066	1.50
**Canu**	contig	527	7.4	1,349	1.62
**Final**	contig	534	9.2	802	2.60

References

1 Mckay, R. J. Sillaginid fishes of the world (family Sillaginidae) : an annotated and illustrated catalogue of the sillago, smelt or Indo-Pacific whiting species known to date. *Fao Fisheries Synopsis* (1992).

2 Mckay, R. J. A revision of the fishes of the family Sillaginidae. *Memoirs of the Queensland Museum* **22**, 1-73 (1985).

3 Nelson, J. S., Grande, T. & Wilson, M. V. H. *Fishes of the World, 5th Edition*. (2016).

4 Shao, K. T. & Chang, K. A revision of the sandborers (Genus: Sillago) of Taiwan. *Bulletin of the Institute of Zoology, Academia Sinica* **17**, 1–11

5 Gao, T. X. *et al.* Description and DNA barcoding of a new sillago species, Sillago sinica (Perciformes: Sillaginidae), from coastal waters of China. *Zoological Studies* **50**, 254-263 (2011).

6 Xiao, J. G., Song, N., Han, Z. Q. & Gao, T. X. Description and DNA barcoding of a new sillago species, sillago shaoi (Perciformes: Sillaginidae), in the Taiwan Strait. *Zoological Studies* **55**, 1-18 (2016).

7 Chevin, L.-M., Lande, R. & Mace, G. M. Adaptation, Plasticity, and Extinction in a Changing Environment: Towards a Predictive Theory. *PLOS Biology* **8**, e1000357, doi:10.1371/journal.pbio.1000357 (2010).

8 Sih, A., Ferrari, M. C. & Harris, D. J. Evolution and behavioural responses to human-induced rapid environmental change. *Evolutionary Applications* **4**, 367-387 (2011).

9 Xiao, J. G. *The taxonomy, phylogeny and biogeography of Sillaginidae in China* Ph.D thesis, Ocean University of China., (2018).

10 Kaga, T., Imamura, H. & Nakaya, K. A new sand whiting, Sillago (Sillago) caudicula , from Oman, the Indian Ocean (Perciformes: Sillaginidae). *Ichthyological Research* **57**, 367-372 (2010).

11 Golani, D., Fricke, R. & Yaron, T. Rehabilitation of Sillago erythraea Cuvier, and redescription of Sillago sihama (Forsskål) (Teleostei: Sillaginidae) from the Red Sea. *Stuttgarter Beiträge zur Naturkunde A, Neue Serie* **4**, 465-471 (2011).

12 Panhwar, S. K., Farooq, N., Qamar, N., Shaikh, W. & Mairaj, M. A new Sillago species (family Sillaginidae) with descriptions of six sillaginids from the northern Arabian Sea. *Marine Biodiversity*, 1-7 (2017).

13 Lescak, E. A. *et al.* Evolution of stickleback in 50 years on earthquake-uplifted islands. *Proceedings of the National Academy of Sciences*, doi:10.1073/pnas.1512020112 (2015).

14 Reid, N. M. *et al.* The genomic landscape of rapid repeated evolutionary adaptation to toxic pollution in wild fish. *Science* **354**, 1305-1308, doi:10.1126/science.aah4993 (2016).

15 Koren, S. *et al.* Canu: scalable and accurate long-read assembly via adaptive k-mer weighting and repeat separation. *Genome Research* **27**, 722 (2017).

16 Chin, C. S. *et al.* Phased diploid genome assembly with single molecule real-time sequencing. *Nature Methods* **13**, 1050 (2016).

17 Andrews, S. FastQC A quality control tool for high throughput sequence data. (2013).

18 Yang, X. *et al.* HTQC: a fast quality control toolkit for Illumina sequencing data. *Bmc Bioinformatics* **14**, 1-4 (2013).

19 Lobo, I. Basic Local Alignment Search Tool (BLAST). *Journal of Molecular Biology* **215**, 403-410 (2008).

20 Flicek, P. *et al.* Ensembl 2014. *Nucleic Acids Research* **42**, D749-D755 (2014).

21 Liu, B. *et al.* Estimation of genomic characteristics by analyzing k-mer frequency in de novo genome projects. *Quantitative Biology* **35**, 62-67 (2013).

22 Chen, S. *et al.* Whole-genome sequence of a flatfish provides insights into ZW sex chromosome evolution and adaptation to a benthic lifestyle. *Nature Genetics* **46**, 253 (2014).

23 Xu, P. *et al.* Genome sequence and genetic diversity of the common carp, Cyprinus carpio. *Nature Genetics* **46**, 1212 (2014).

24 Wang, Y. *et al.* The draft genome of the grass carp (Ctenopharyngodon idellus) provides insights into its evolution and vegetarian adaptation. *Nature Genetics* **47**, 625-631 (2015).

25 Kajitani, R. *et al.* Efficient de novo assembly of highly heterozygous genomes from whole-genome shotgun short reads. *Genome Research* **24**, 1384-1395 (2014).

26 Fu, X. *et al.* Long-read sequence assembly of the firefly Pyrocoelia pectoralis genome. *Gigascience* **6**, 1-7 (2017).

27 Conte, M. A., Gammerdinger, W. J., Bartie, K. L., Penman, D. J. & Kocher, T. D. A high quality assembly of the Nile Tilapia (Oreochromis niloticus) genome reveals the structure of two sex determination regions. *Bmc Genomics* **18**, 341 (2017).

28 Zhang, J. *et al.* Genome puzzle master (GPM): an integrated pipeline for building and editing pseudomolecules from fragmented sequences. *Bioinformatics* **32**, 3058-3064 (2016).

29 Pryszcz, L. P. & Gabaldón, T. Redundans: an assembly pipeline for highly heterozygous genomes. *Nucleic Acids Research* **44**, e113-e113 (2016).

30 Ichikawa, K. *et al.* Centromere evolution and CpG methylation during vertebrate speciation. *Nature Communications* **8** (2017).

31 Conte, M. A., Gammerdinger, W. J., Bartie, K. L., Penman, D. J. & Kocher, T. D. A high quality assembly of the Nile Tilapia (Oreochromis niloticus) genome reveals the structure of two sex determination regions. *Bmc Genomics* **18**, 341 (2017).

32 Parra, G., Bradnam, K. & Korf, I. CEGMA: a pipeline to accurately annotate core genes in eukaryotic genomes. *Bioinformatics* **23**, 1061 (2007).

33 Simão, F. A., Waterhouse, R. M., Ioannidis, P., Kriventseva, E. V. & Zdobnov, E. M. BUSCO: assessing genome assembly and annotation completeness with single-copy orthologs. *Bioinformatics* **31**, 3210 (2015).

34 Li, H. & Durbin, R. Fast and accurate short read alignment with Burrows–Wheeler transform. *Bioinformatics* **25**, 1754-1760 (2009).

35 Benson, G. Tandem repeats finder: a program to analyze DNA sequences. *Nucleic Acids Research* **27**, 573 (1999).

36 Bao, W., Kojima, K. K. & Kohany, O. Repbase Update, a database of repetitive elements in eukaryotic genomes. *Mobile Dna* **6**, 11 (2015).

37 Chen, N. Using RepeatMasker to identify repetitive elements in genomic sequences. *Current Protocols in Bioinformatics* **Chapter 4**, Unit 4.10 (2004).

38 Stanke, M. *et al.* AUGUSTUS: ab initio prediction of alternative transcripts. *Nucleic Acids Research* **34**, 435-439 (2006).

39 Gertz, E. M. *et al.* Composition-based statistics and translated nucleotide searches: Improving the TBLASTN module of BLAST. *Bmc Biology* **4**, 41 (2006).

40 Birney, E., Clamp, M. & Durbin, R. GeneWise and Genomewise. *Genome Research* **14**, 988 (2004).

41 Trapnell, C., Pachter, L. & Salzberg, S. L. TopHat: discovering splice junctions with RNA-Seq. *Bioinformatics* **25**, 1105-1111 (2009).

42 Ghosh, S. & Chan, C. K. K. Analysis of RNA-Seq data using TopHat and Cufflinks. *Methods in Molecular Biology* **1374**, 339 (2016).

43 Campbell, M. S., Holt, C., Moore, B. & Yandell, M. Genome Annotation and Curation Using MAKER and MAKER-P. *Current Protocols in Bioinformatics* **48**, 4.11.11 (2014).

44 Harris, M. A. *et al.* The Gene Ontology (GO) database and informatics resource. *Nucleic Acids Research* (2004).

45 Ogata, H. *et al.* KEGG: Kyoto Encyclopedia of Genes and Genomes. *Nucleic Acids Research* **27**, 29-34 (2000).

46 Conesa, A. *et al.* Blast2GO: a universal tool for annotation, visualization and analysis in functional genomics research. *Bioinformatics* **21**, 3674 (2005).

47 Lowe, T. M. & Eddy, S. R. tRNAscan-SE: a program for improved detection of transfer RNA genes in genomic sequence. *Nucleic Acids Research* **25**, 955-964 (1997).

48 Griffithsjones, S., Bateman, A., Marshall, M., Khanna, A. & Eddy, S. R. Rfam: an RNA family database. *Nucleic Acids Research* **31**, 439 (2003).

49 Nawrocki, E. P. & Eddy, S. R. Infernal 1.1: 100-fold faster RNA homology searches. *Bioinformatics* **29**, 2933-2935 (2013).

50 Thompson, J. D., Gibson, T. & Higgins, D. G. Multiple sequence alignment using ClustalW and ClustalX. *Current protocols in bioinformatics*, 2.3. 1-2.3. 22 (2002).

51 Guindon, S., Dufayard, J. F., Hordijk, W., Lefort, V. & Gascuel, O. PhyML: Fast and Accurate Phylogeny Reconstruction by Maximum Likelihood. **9**, 384-385 (2009).

52 Yang, Z. & Rannala, B. Bayesian estimation of species divergence times under a molecular clock using multiple fossil calibrations with soft bounds. *Molecular Biology & Evolution* **23**, 212-226 (2006).

53 Hedges, S. B., Marin, J., Suleski, M., Paymer, M. & Kumar, S. Tree of life reveals clock-like speciation and diversification. *Molecular Biology & Evolution* **32**, 835-845 (2015).

54 Xu S, Xiao S, Zhu S, Zeng X, Luo J, Liu J et al. Supporting data for "A draft genome assembly of the Chinese sillago (Sillago sinica), the first reference genome for Sillaginidae fishes" GigaScience Database 2018. http://dx.doi.org/10.5524/100490

Supplementary Figures

Click here to access/download
Supplementary Material
SI_Figures.docx

Supplementary Tables

Click here to access/download
Supplementary Material
SI_Table.docx
