# Supplementary material for: A draft genome assembly of the Chinese sillago (Sillago sinica), the first reference genome for Sillaginidae fishes
Source: Gigascience. 2018 Sep 10;7(9):giy108. doi: 10.1093/gigascience/giy108 (PMC6143730; doi:10.1093/gigascience/giy108)
Supplement: Reviewer_1_Original_Submission_(attachment).pdf [file giy108_reviewer_1_original_submission_(attachment).pdf]

High-quality genome assembly of the Chinese sillago (*Sillago sinica*), the first high-quality reference genome for Sillaginidae fishes

Shengyong Xu[1*], Shijun Xiao[2*], Shilin Zhu[2], Xiaofei Zeng[2], Jing Luo[3], Tianxiang Gao[1,#], Nansheng Chen[4,5,#]

[1] Fishery College, Zhejiang Ocean University, Zhoushan, Zhejiang, China
[2]Wuhan Frasergen Bioinformatics Co., Ltd., Wuhan, Hubei, China
[3]School of Life Sciences, Yunnan University,Kunming, Yunnan,China
[4]Institute of Oceanology, Chinese Academy of Sciences, Qingdao, Shandong, China
[5]Department of Molecular Biology and Biochemistry, Simon Fraser University, Burnaby, Canada

Abstract

Background

Sillaginidae, also known as smelt-whitings, is a family of benthic coastal marine fishes in theIndo-West Pacific that have high ecological and economic importance. Many Sillaginidae species, including the Chinese sillago (*Sillago sinica*) are recently described in China, providing us with valuable materials to analyze genetic diversification of the family Sillaginidae. Herein, we constructed a high-quality reference genome for the Chinese sillago, with the aim to setup a platform for comparative analysis of all species in this family.

Findings

Using the single-molecule real-time DNA sequencing platform PacBio Sequel, we generated ~27.3 Gb genomic DNA sequences for the Chinese sillago. We reconstructed a genome assembly of 534 Mb using a strategy that takes advantage of complementary strengths of two genome assembly programs Canu and FALCON. The genome size was consistent with the estimated genome size based on k-mer analysis. The genome assembly reached a remarkable high level of continuity with contig N50 length of 2.6 Mb, and the entire Chinese sillago genome consists of only 802 contigs. We annotated 22,122 protein-coding genes in the Chinese sillago genomes using *de novo* method and with RNA-seq data and homologies to other teleosts. According to the phylogenetic analysis using protein-coding genes, Chinese sillago was closely related to *Larimichthys Crocea* and *Dicentrarchus labrax*, and Chinese sillago diverged from their ancestor around 69.5 - 82.6 million years ago.

Conclusions

We have built a high-quality genome assembly for the Chinese sillago using long reads generated with PacBio sequencing technologies, which is the first reference genome for Sillaginidae species. This genome assembly sets a stage for comparative analysis of the diversification and adaptation of fishes in Sillaginidae.

Key Words: Sillaginidae, Chinese sillago, PacBio sequencing, Canu, FALCON, genetic diversification

Data description

The fish family Sillaginidae consists of demersal marine fishes commonly known as sand whitings or sand borers[1] that inhabit inshore waters throughout the Indo-West Pacific[2,3]. As ecologically and commercially important marine organisms, Sillaginidae species play vital important roles in the commercial fisheries of Pakistan, Australia, China, Malaysia, Thailand and Philippines[1,4]. Owing to similar phenotypic characteristics, delineation and identification of Sillaginidae species often confuse the taxonomists. Additionally, rapid environment changes resulting from anthropogenic activities can force Sillaginidae species to adapt to diversifying situations, leading to further diversification and speciation. Numerous cryptic lineages were identified in *S. sihama* complex by using phenotypic traits and molecular markers in the Northwestern Pacific. For example, five recently identified Sillago species were misidentified as *S. sihama*[5-9]. Therefore, it is essential to investigate Sillaginidae species at the genetics level to identify molecular features for accurate characterization of different species, and for understanding rapid genetic diversification and speciation. Among Sillaginidae species, the Chinese sillago *Sillago sinica* (Figure 1) is one of the most recently identified Sillaginidae species in the Northwestern Pacific[6]. Due to their phenotypic similarity, *S. sinica* was previously misidentified as *S. sihama*. However, these two fish species are different because *S. sinica* inhabits a cold-temperate environment while *S. sihama* inhabits a warm-temperate environment. It is thus essential to sequence the genome of *S. sinica*, which will improve taxonomy, and may help to reveal insights into evolutionary history of Sillaginidae species and the role of environment changes in rapid genetic diversification and speciation.

Here we present a high-quality reference genome assembly for *S.sinica* constructed using long reads generated by the PacBio DNA sequencing platform Sequel, and using a genome assembly strategy taking advantage of two genome assemblers Canu[10] and FALCON[11]. This genome assembly of the Chinese sillago *Sillago sinica* is the first genome constructed for the family Sillaginidae. The completeness and continuity of the genome provided high quality genomic resources for studies on evolutionary history of the rapid speciation processes of Sillaginidae species.

Sample and DNA extraction

To obtain enough high-quality genomic DNA for PacBio Sequel sequencing (Pacific Biosciences of California, Menlo Park, CA, USA), we collected fresh muscle tissue from a Chinese sillago fish in Zhoushan city, Zhejiang province. The sample was quickly frozen in liquid nitrogen for one hour before storing at −80°C. Genomic DNA was extracted using standard phenol/chloroform extraction protocol. The integrity of genomic DNA molecules

was checked using agarose gel electrophoresis, which showed a main band around 20 kb, indicating high-quality for PacBio Sequel platform.

Genome size estimation

To estimate the Chinese sillago genome size, we also sequenced the genomic DNA using Illumina DNA sequencing technologies. Five paired-end libraries were constructed with insert sizes of 250 base pairs (bp), 300 bp, 500 bp, 800 bp, 2 kb and generated a total of ~42 Gb sequence data (Table 1, SI Table 1) on Illumina HiSeq X Ten platform (Illumina Inc., San Diego, CA, USA). Raw reads were analyzed using FastQC[12] and then filtered using HTQC[13]. Low

quality bases and reads were filtered in the following filtering steps:1) Removing adaptor sequences introduced during sequencing library construction; 2) Removing read pairs if the average base quality was lower than 20 for any of the two ends; 3) Trimming ambiguous or low quality fragments at two ends of reads within a window size of 5 bp and an average quality threshold of 20; 4) Removing read pairs if any of the two reads had a read length shorter than 75. A single peak around 45% were identified in GC distribution (SI Figure 1) for cleaned sequencing reads. After searching against the non-redundant nucleotide (nt) database with BLASTN[14], we found that the best hits were enriched for closely related fish species[15], including *Oryzias latipes*, *Larimichthys corcea*, *Cyprinus carpio* and *Dicentrarchus labrax*.

We estimated the genome size of the Chinese sillago by analyzing the 17-mer depth distribution[16] using the following equation:

$$G = N_{17\text{-mer}} / D_{17\text{-mer}}$$

The $N_{17\text{-mer}}$ is the total number of 17-mers, and $D_{17\text{-mer}}$ denotes the peak frequency of 17-

mers. For our data, $N_{17\text{-mer}}$ was 37,811,957,476 and $D_{17\text{-mer}}$ was 66, suggesting an estimated genome size of 524 Mb. Meanwhile, we observed a heterozygous and a repeat peak (SI Figure 2), with an estimated heterozygosity of 0.76% (1 SNP/ 100nt) and a repeat content of 12.7% for the Chinese sillago individual used in this work. The heterozygosity of our sample was noticeably higher than other fish species in previous genome studies[17-19], partly because the Chinese sillago sample used in this project was collected directly from the wild environment without further artificial inbreeding. Pilot assembly using the Illumina data and the assembly program Platanus package[20] produced a 624 Mb genome assembly with a contig N50 length of 3.2 kb (Table 2). This genome assembly was of low-quality due to its high genomic heterozygosity.

Genome assembly using PacBio long reads

Because of the high heterozygosity for the Chinese sillago, we first used FALCON[11] for genome assembly. With the parameter of length_cutoff set at 10 kb and pr_length_cutoff at 8 kb, we produced a 546 Mb genome assembly for the Chinese sillago, which agreed well with the estimated genome size in 17-mer analysis (above). The

genome assembly consisted of only 2,066 contig with a N50 length of 1.5 Mb (Table 2).[10] Meanwhile, we also applied Canu (v1.4) to assemble the genome with the

We prepared two 20 kb genomic DNA libraries, which were sequenced using PacBio Sequel using five SMRT cells, generating 27.3 Gb raw DNA reads (Table 1, SI Table 2).

After removing adaptor sequences, we obtained 3.4 million subreads (totally 27.2 Gb) with a contig N50 length of 12.96kb (SI Table 3, SI Figure 3).

CorrectedErrorRate parameter set at 0.052. As a result, we obtained a second Chinese

sillago genome of 527 Mb, with 1,349 contigs and contig N50 of 1.62 Mb (Table 2). Thus, both assemblies have similar genome sizes and excellent continuity, suggesting good quality of both genome assemblies. We then used Genome Puzzle Master (GPM)[21] to merge the two genome assemblies into an integrated genome by tracking the overlapping relationships between contigs of the two genome assemblies, and applied Redundans[22] (v0.13c) to remove the sequence redundancy. The resulting genome assembly was further polished using NGS data, which were used in the genome survey analysis above. The contig N50 length of the final 534 Mb Chinese sillago genome assembly reached 2.6 Mb (Table 2). The contig N50 of the Chinese sillago is much higher than those of previous fish genome assemblies constructed using NGS DNA sequencing technologies, and is comparable with those of recently reported model fish species[23,24]. (Figure 2).

Genome quality evaluation

To validate the completeness of the Chinese sillago genome assembly, we subjected the sequences to CEGMA[25] and BUSCO[26] evaluation. More than 96% of core eukaryotic genes were successfully identified in the Chinese sillago genome in both CEGMA (SI Table 4) and BUSCO (SI Table 5) analyses, implying high completeness of the Chinese sillago genome

assembly [96% identified but what % are full length?].

To further evaluate the accuracy of the Chinese sillago genome assembly, we aligned the NGS-based short reads from whole-genome sequencing data against the genome assembly using BWA[27]. We found that 98.4% of the reads were reliably aligned to the genome assembly, and 95.8% of the reads were properly aligned to the genome with their mates [how does this correlate with the heterozygosity measurement? Pretty well by the look]. The insertion length distribution for sequencing library of 250 bp, 300 bp, 500 bp, 800 bp, 2 kb exhibited a single peak around the sequencing library length chosen (SI Figure 4), illuminating the high quality of the genome assembly. Using genomic homozygous mutations detected from the NGS data, we estimated the genome accuracy at the base level reached 99.997%.

Repeat annotation

We annotated repetitive elements in the Chinese sillago genome using Tandem Repeat Finder[28]. To identify transposon elements (TE), RepeatModeler (http://www.repeatmasker.org/RepeatModeler.html) was used firstly to identify de novo repeat types in the genome. The Repbase database[29] of known repeats and a de novo repeat library generated by RepeatModeler were used. The TEs in the Chinese sillago genome were then identified by mapping to the library using the software RepeatMasker[30]. We found that tandem repeat content in Chinese sillago (4.69%) was much higher than those in *Gasterosteus aculeatus* (2.03%), *Larimichthys corcea* (2.7%), *Oryzias latipes* (0.92%) and *Dicentrarchus labrax*(2.8%). However, the content of TEs (12.86%) of the Chinese sillago was lower than those of the above fish species (SI Figure 5, SI Table 6), leading to an overall lower content of repetitive sequences in the Chinese sillago genome, which might be a reason for the relatively small genome size of Chinese sillago.

RNA preparation and sequencing

We also sequenced, using Illumina sequencing technologies, cDNA libraries prepared from the same Chinese sillago fish individual used for genome annotation . Tissues of ocular, skin, muscle, gonadal, intestinal, liver, kidney, blood, gall and air bladder tissues were collected and RNAs were extracted with TRIZOL Reagent (Invitrogen, USA). RNAs were then balanced mixed for the sequencing. The absorbance of 1.90 at 260 nm/280 nm and the RIN of 9.1 were obtained for the purified RNA sample by Nanodrop ND-1000 spectrophotometer (LabTech, USA) and 2100 Bioanalyzer (Agilent Technologies, USA), respectively.

173
177
182
189
162
163
168
174 175

According to the protocol suggested by the manufacturer, one microgram of RNA was reverse transcribed using Clontech SMARTer cDNA synthesis kit, and was further fragmented using divalent cations for NGS sequencing. The paired-end library was prepared following the manual of the Paired-End Sample Preparation Kit (Illumina Inc., San Diego, CA, USA). Finally, the library with an insert length of 300 bp was sequenced by Illumina HiSeq X Ten in 150PE mode (Illumina Inc., San Diego, CA, USA). As a result, we obtained ~10.4 Gb high-quality transcriptome data from RNA-seq (Table 1, SI Table 1).

Gene and functional annotation

To annotate genes in the Chinese sillago genome, gene prediction was performed with de novo, homology-based and transcriptome sequencing-based methods. We first used Augustus[31] to predict protein-coding genes in the Chinese sillgo genome. Then, protein sequences of closely related fish species, including *Danio rerio*, *Dicentrarchus labrax*, *Gasterosteus aculeatus*, *Larimichthys corcea*, *Oryzias latipes*, *Takifugu rubripes* and *Gadus morhua*, were downloaded from Ensembl[15] and aligned against to the Chinese sillago genome using TBLASTN software[32]. GeneWise[33] was then used to define gene models. We also used NGS transcriptome short reads aligned upon the Chinese sillago genome using the TopHat package[34], and the gene structures were predicted using Cufflinks[35]. All gene models were then integrated using MAKER[36] to obtain a consensus gene set (SI Figure 6). Altogether, we annotated 22,122 protein-coding genes in the Chinese sillago genome. The gene number, gene length distribution, CDS length distribution, exon length distribution and intron length distribution were all comparable with those in other teleost fish species (SI Figure 7, SI Table 7).

To obtain functional annotation of the protein-coding genes in the Chinese sillago

genome, we searched the NCBI non-redundant protein (nr), non-redundant nucleotide (nt),[14] [37] and Swissprot database using local BLASTX and BLASTN programs with an e-value threshold of 1e-5 . We then searched the Gene ontology (GO) and Kyoto Encyclopedia of Genes and Genomes (KEGG)[38] pathway databases using the software Blast2GO[39]. As a result, most (21,768) of the 22,122 genes were annotated by at least one database, representing 98.4% of the total genes (SI Figure 8, SI Table 8). We also annotated four types of non-coding RNAs (microRNAs, transfer RNAs, ribosomal RNAs, and small nuclear RNAs) using tRNAscan-SE[40] [41] and the Rfam database [Was Rfam searched using Infernal? This needs to be put explicitly as it could be interpreted as search by sequence similarity]

including Dicentrarchus labrax, Larimichthys corcea, Astyanax mexicanus, Danio rerio,

families were obtained by clustering of homologous gene sequences using H-scores in

To generate the phylogenetic relationship of Chinese sillago with other fish species, the

(SI Table 9).

Gene family identification

In order to identify gene families among fish species in this work, proteins of the longest transcripts of each individual genes from the Chinese sillago and other fish species, Gadusmorhua, Gasterosteus aculeatus, Lepisosteus oculatus, Oryzias latipes, Takifugu rubripes, Xiphophorus maculates and Callorhynchus milii, were aligned to each other with BLASTP[14] programs with an e-value threshold of 1e-5. The HSP segments were concatenated by Solar, and H-scores were calculated from Bit-score. At last, gene Hcluster_sg software. As a result, 15,022 gene families were constructed for the Chinese sillago (Figure 3).

Phylogenetic analysis for Chinese sillago and fishes with public genome

coding sequences of single-copy gene families among all species were extracted and aligned with the guidance of protein alignment from ClustalW program[42] and the alignment were concatenated as a single data set. The maximum-likelihood method implemented in

medaka from the TimeTree database[45]. According to the phylogenetic analysis, Chinese

the PhyML package[43] with the JTT+G+F model were used to construct the phylogenetic tree from the super-alignment of the coding sequences. The MCMCtree program in the PAML package was used to determine divergence times with the approximate likelihood method[44] and a molecular clock data from the divergence time between zebrafish and sillago were clustered together with Larimichthys Crocea and Dicentrarchus labrax, which is consistent with the fish species taxonomy. Chinese sillago diverged from the common ancestor with Larimichthys Crocea and Dicentrarchus labrax around 69.5-82.6 million years ago. (Figure 4)

Conclusion

Using long reads from the third-generation PacBio Sequel sequencing platform, we successfully assembled the genome of the Chinese sillago, which represents the first high-quality genome of all species in Sillaginidae species. The 534 Mb Chinese sillago genome assembly consists of only 802 contigs with contig N50 length of 2.6 Mb. The contig N50 is remarkably longer than those of most fish genome assemblies, and is comparable with those of recently reported model fish species. The genome base accuracy reached 99.997%. We annotated 22,122 protein-coding genes in the Chinese sillago genome assembly. We found that Chinese sillago diverged from the common ancestor of Larimichthys Crocea and Dicentrarchus labrax around 69.5 - 82.6 million years ago. The genome assembly, together with gene annotation and transcriptome data generated in this work provided a valuable resource for research on the phylogenetic and adaption investigation of Sillaginidae family, and contribute positively to large-scale projects such as Genome 10K[46].

Ethics Statement

272

274

281

283

285

This study was approved by the Animal Care and Use committee of Fishery College of Zhejiang Ocean University. All participates consent the study under the 'Ethics, consent and permissions' heading. All participants consent to publish the work under the 'Consent to publish' heading.

Availability of supporting data

Competing interests

The authors declare that they have no competing interests.

Supporting data and materials are available in the GigaScience GigaDB database, with the raw genome sequences deposited in the SRA under the bioproject number PRJNA437933.

Funding

This study was supported by a grant from the National Natural Science Foundation of China (No.41776171; No.31572227; No.31602207), Scientific Startup Foundation of Zhejiang Ocean University (No.Q1505) and the Open Foundation from Fishery Sciences in the First-Class Subjects of Zhejiang (No.20160001).

Author Contributions

TXG and NSC conceived the project. SYX collected the samples and extracted the genomic DNA. SJX, SLZ and XFZ performed the genome assembly and data analysis. TXG, NSC and SJX, JL wrote the paper.

Figure Legends

Figure 1. A representative individual of the Chinese sillago.

Figure 2. Comparing genome assemblies between Chinese sillago and other fish species. Top 20 public genomes ordered by contig N50 lengths (A) or contig numbers (B).

Figure 3. Gene family comparison between Chinese sillago and other fish species.

Figure 4. The phylogenetic relationship of Chinese sillago with other fish.

Tables

Table 1 Summary of sequence data from Sillago sinica

Type	Method	Library size (bp)	Data size (Gb)	Read N50 (bp)
DNA	HiSeq 2000	250, 300, 500, 800, 2000	42.3	150
DNA	PacBio Sequel	20,000	27.3	12,957
RNA	HiSeq 2000	250	10.5	150
