# Supplementary material for: A draft genome assembly of the Chinese sillago (Sillago sinica), the first reference genome for Sillaginidae fishes
Source: Gigascience. 2018 Sep 10;7(9):giy108. doi: 10.1093/gigascience/giy108 (PMC6143730; doi:10.1093/gigascience/giy108)
Supplement: Supplemental Files [file giy108_supplemental_files.zip › SI_Figures.docx]

Supporting Information Figures for

**A draft genome assembly of the Chinese sillago (*Sillago sinica*), the first reference genome for Sillaginidae fishes**


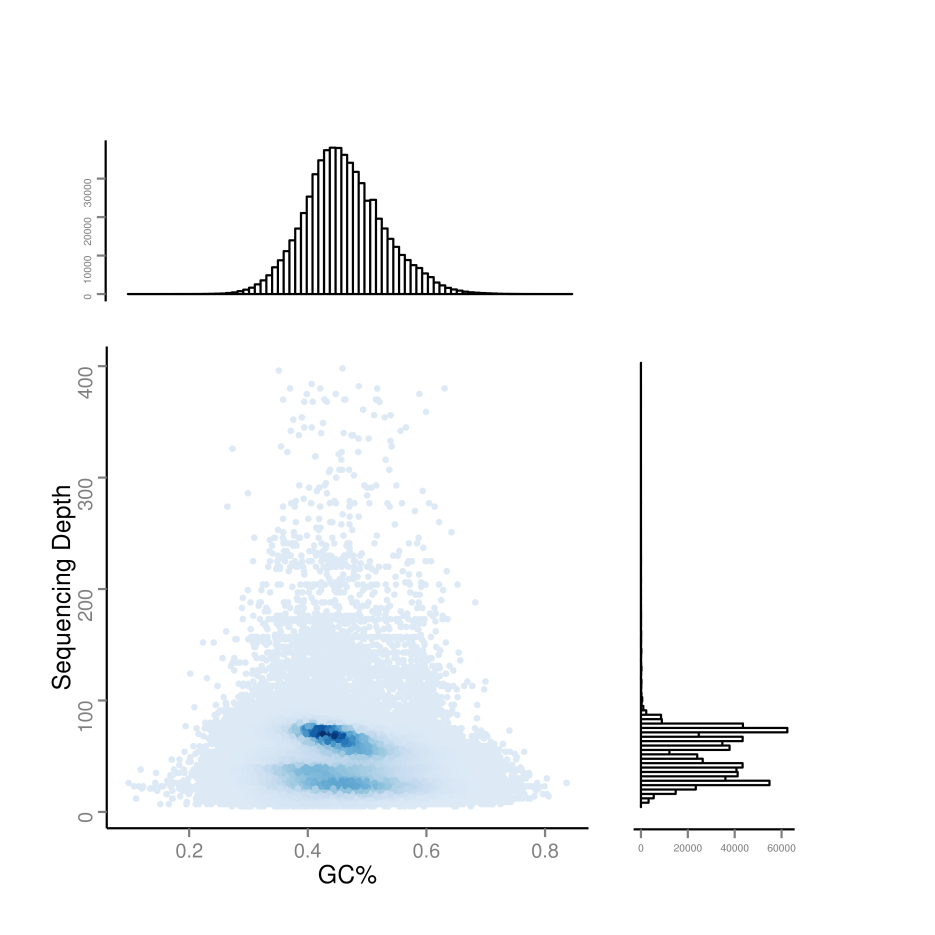


SI Figure 1. GC and sequence distribution for *Sillago sinica* genome assembly


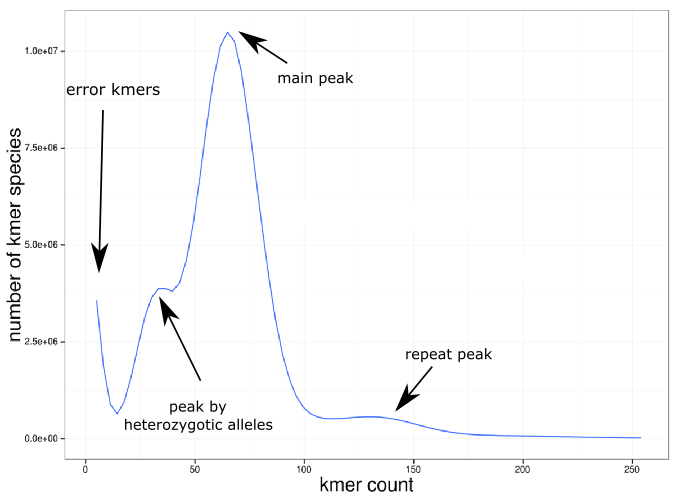


SI Figure 2. 17-mer analysis for genome size estimation


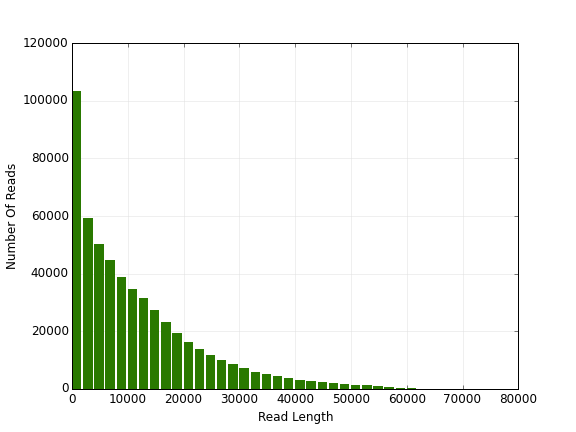


SI Figure 3. Polymerase length distribution from PacBio SEQUEL sequencing


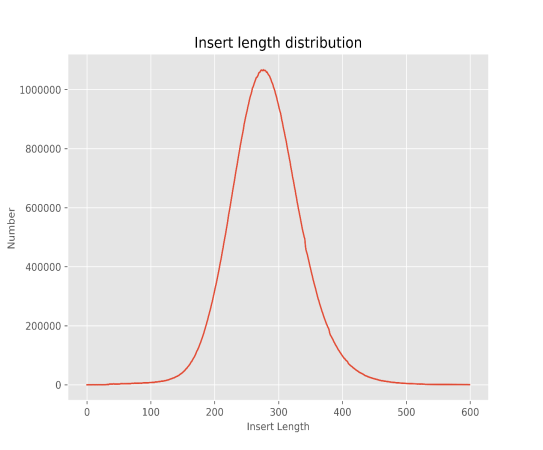

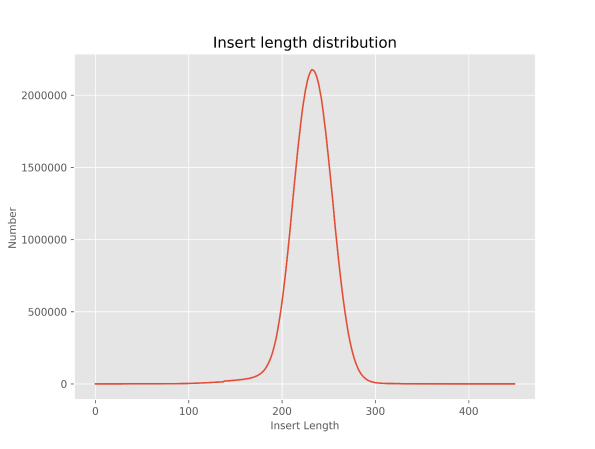


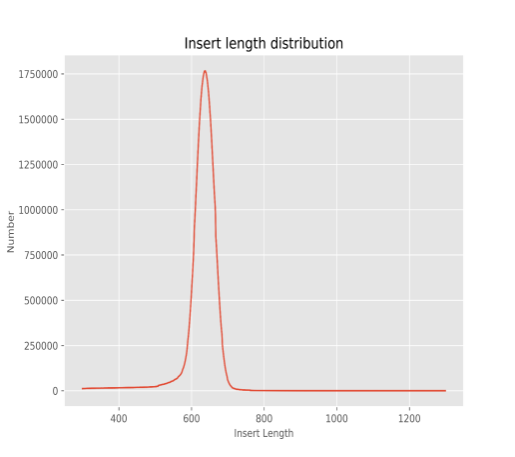

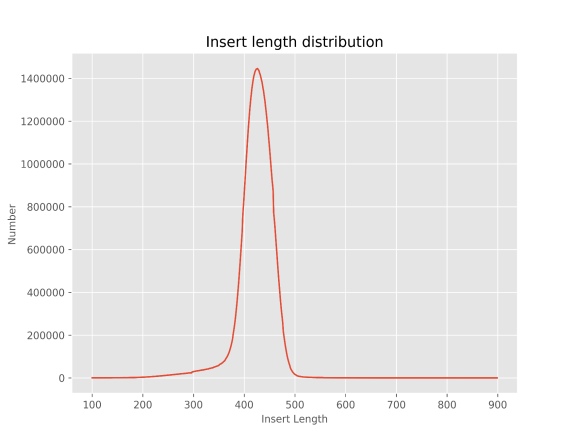
 PE250bp PE300bp

PE500bp PE800bp


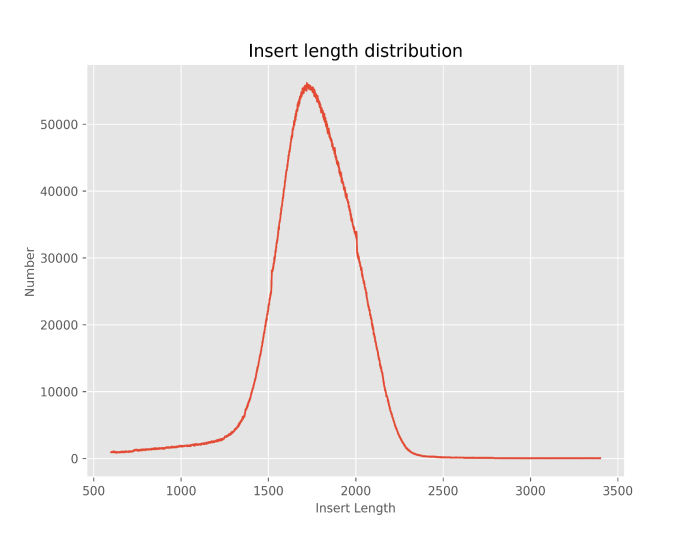


MP2kb

SI Figure 4. Genome sequence validation using NGS reads from libraries with various insertion length


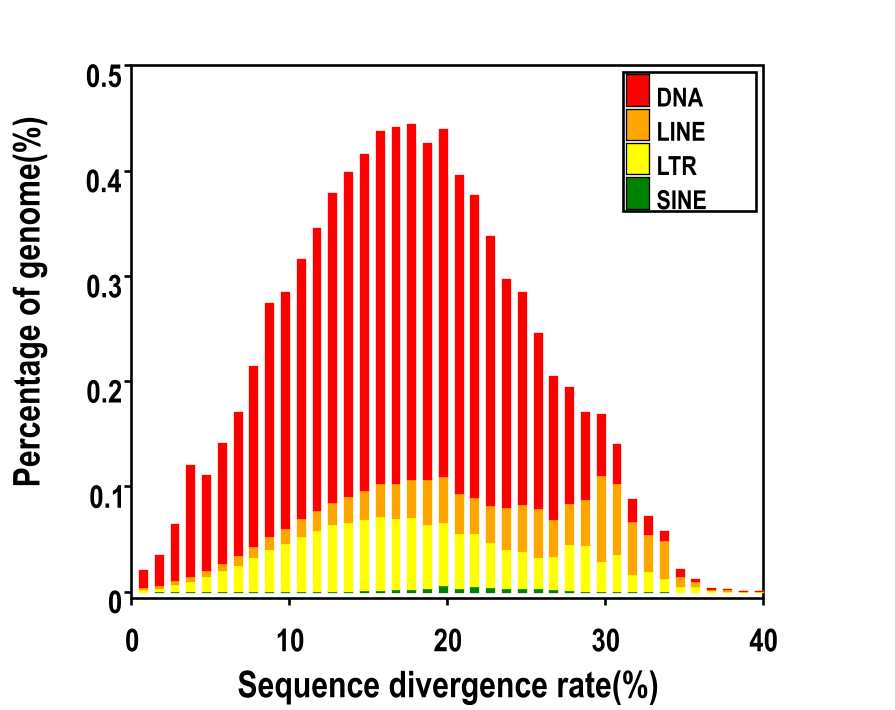


SI Figure 5. Sequence divergence rate of TEs in Chinese sillago against to RepBase


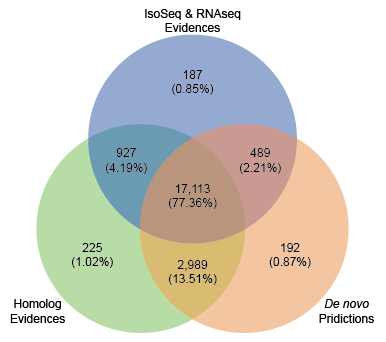


SI Figure 6. Venn plot for gene prediction using different method


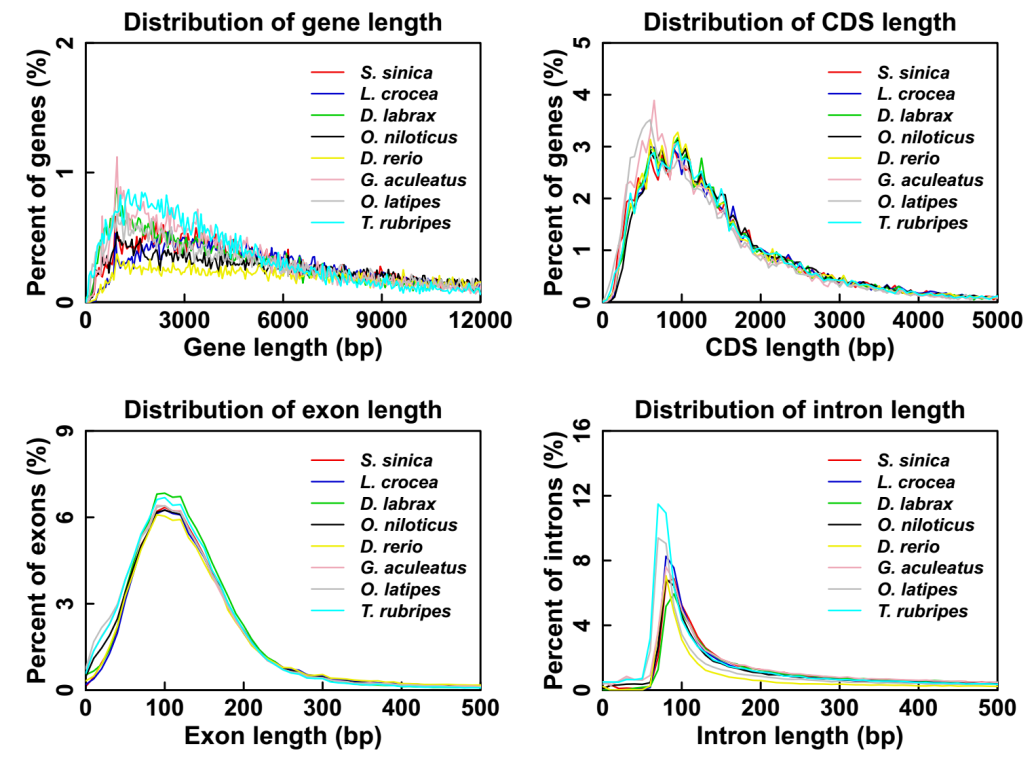


SI Figure 7. Chinese sillago gene structure comparison to other teleost


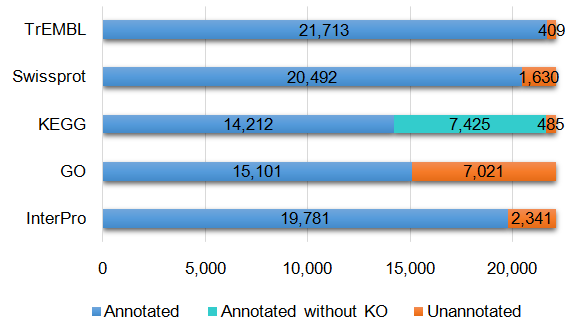


SI Figure 8. Functional annotation of genes predicted in the Chinese sillago genome
