# Supplementary material for: A draft genome assembly of the Chinese sillago (Sillago sinica), the first reference genome for Sillaginidae fishes
Source: Gigascience. 2018 Sep 10;7(9):giy108. doi: 10.1093/gigascience/giy108 (PMC6143730; doi:10.1093/gigascience/giy108)
Supplement: Supplemental Files [file giy108_supplemental_files.zip › SI_Table.docx]

Supporting Information Tables for

**A draft genome assembly of the Chinese sillago (*Sillago sinica*), the first reference genome for Sillaginidae fishes**

SI Table 1. DNA and RNA sequencing for Chinese sillago based on NGS

| **Type** | **Reads number** | **Base(bp)** | **Q20(%)** | **Q30(%)** | **GC Content(%)** |
| --- | --- | --- | --- | --- | --- |
| DNA | 141,202,110 | 42,360,633,000 | 97.56;93.98 | 94.14;86.92 | 44.89;45.13 |
| RNA | 69,856,504 | 10,478,475,600 | 98.29;93.66 | 95.50;86.88 | 52.58;52.79 |

SI Table 2. Species hit table by searching NGS sequencing reads to the NCBI NT database

| **Species** | **number** | **Percentage (%)** |
| --- | --- | --- |
| *Oryzias latipes* | 1,773 | 8.87 |
| *Larimichthys crocea* | 1,226 | 6.13 |
| *Cyprinus carpio* | 1,208 | 6.04 |
| *Dicentrarchus labrax* | 1,176 | 5.88 |
| *Danio rerio* | 570 | 2.85 |

SI Table 3. *K*mer-based method to estimate the genome characters

| ***K*mer** | **Genome size (Mb)** | **Heterozygosity**  **(%)** | **Repeat content**  **(%)** |
| --- | --- | --- | --- |
| 17 | 524 | 0.76 | 12.8 |
| 21 | 519 | 0.75 | 12.9 |
| 27 | 523 | 0.66 | 11.3 |

SI Table 4. Polymerase statistics for DNA genome sequencing based on PacBio

| **Sample** | **Polymerase read bases (bp)** | **Polymerase read number** | **Average polymerase read length (bp)** | **Polymerase read N50 (bp)** | **Insert length (bp)** |
| --- | --- | --- | --- | --- | --- |
| **XI2-D 1** | 6,267,937,183 | 535,941 | 11,695 | 19,750 | 9,255 |
| **XI2-D 2** | 5,295,467,795 | 516,752 | 10,248 | 18,750 | 8,120 |
| **XI2-D 3** | 4,628,024,442 | 540,064 | 8,569 | 15,250 | 7,321 |
| **XI2-D 4** | 5,461,978,538 | 448,361 | 12,182 | 20,250 | 9,839 |
| **XI2-D 4** | 5,643,382,158 | 447,362 | 12,615 | 21,250 | 9,500 |
| **total** | 27,296,790,116 | 2,488,480 |  |  |  |

SI Table 5. Subread statistics for DNA genome sequencing based on PacBio

| **Sample** | **Total bases (bp)** | **Subread number** | **Average subread length (bp)** |
| --- | --- | --- | --- |
| XI2-D 1 | 6,257,543,640 | 751,177 | 8,330 |
| XI2-D 2 | 5,285,891,372 | 714,266 | 7,400 |
| XI2-D 3 | 4,620,633,393 | 689,326 | 6,703 |
| XI2-D 4 | 5,453,788,678 | 619,495 | 8,804 |
| XI2-D 4 | 5632,596,624 | 664,904 | 8,471 |
| total | 27,250,453,707 | 3,439,168 |  |

SI Table 6. CEGMA result to analysis genome completeness for Chinese sillago

|  | **Proteins** | **%Completeness** | **Proteins** | **%Completeness** |
| --- | --- | --- | --- | --- |
|  | **completed** | | **partial** | |
| Total | 239 | 96.37 | 246 | 99.19 |
| Group 1 | 65 | 98.48 | 66 | 100.00 |
| Group 2 | 55 | 98.21 | 56 | 100.00 |
| Group 3 | 56 | 91.80 | 59 | 96.72 |
| Group 4 | 63 | 96.92 | 65 | 100.00 |

SI Table 7. BUSCO result to analysis genome completeness for Chinese sillago

|  | **Proteins** | **Percentage(%)** |
| --- | --- | --- |
| Complete BUSCOs | 4430 | 96.64 |
| Complete Single-Copy BUSCOs | 4256 | 92.84 |
| Complete Duplicated BUSCOs | 174 | 3.80 |
| Fragmented BUSCOs | 85 | 1.85 |
| Missing BUSCOs | 69 | 1.51 |
| **Total BUSCO groups searched** | 4584 | 100.00 |

SI Table 8. Repeat annotation in Chinese sillago genome

| **Species** | ***Sillago sinica*** | | ***Gasterosteus aculeatus*** | | ***Larimichthys crocea*** | | ***Oryzias latipes*** | | ***Dicentrarchus labrax*** | |
| --- | --- | --- | --- | --- | --- | --- | --- | --- | --- | --- |
|  | **Length**  **(bp)** | **Percent**  **(%)** | **Length**  **(bp)** | **Percent**  **(%)** | **Length**  **(bp)** | **Percent**  **(%)** | **Length**  **(bp)** | **Percent**  **(%)** | **Length**  **(bp)** | **Percent**  **(%)** |
| Repeats | 89,406,631 | 16.73 | 74,414,211 | 16.12 | 126,503,655 | 18.63 | 248,699,894 | 28.59 | 147,870,759 | 21.88 |
| TEs | 68,695,559 | 12.86 | 65,053,183 | 14.10 | 108,725,187 | 16.01 | 240,824,277 | 27.69 | 128,146,276 | 18.96 |
| SINE | 1,061,105 | 0.20 | 1,923,029 | 0.42 | 4,267,240 | 0.63 | 7,715,378 | 0.89 | 3,611,675 | 0.53 |
| LINE | 10,967,581 | 2.05 | 12,743,463 | 2.76 | 18,759,105 | 2.76 | 44,327,731 | 5.10 | 18,401,365 | 2.72 |
| LTR | 13,615,771 | 2.55 | 13,144,595 | 2.85 | 12,166,781 | 1.79 | 12,291,803 | 1.41 | 6,069,419 | 0.90 |
| DNA | 52,895,483 | 9.90 | 21,880,271 | 4.74 | 35,549,002 | 5.24 | 87,546,101 | 10.06 | 49,008,386 | 7.25 |
| Unclassified | 1,580,921 | 0.30 | 15,361,825 | 3.33 | 37,983,059 | 5.59 | 88,943,264 | 10.23 | 51,055,431 | 7.55 |
| Tandem repeats | 25,047,581 | 4.69 | 9,360,371 | 2.03 | 18,335,311 | 2.70 | 7,963,451 | 0.92 | 18,976,620 | 2.81 |

SI Table 9. Protein-coding gene prediction in Chinese sillago genome

| Gene set | | Protein  coding  gene  number | Average gene length (bp) | Average CDS length (bp) | Average exon per gene | Average exon length (bp) | Average intron length (bp) |
| --- | --- | --- | --- | --- | --- | --- | --- |
| **De novo** | **AUGUSTUS** | 23,142 | 10738.33 | 1550.12 | 9.01 | 172.01 | 1146.85 |
| **Homolog** | **Genscan** | 29,523 | 13161.99 | 1569.52 | 9.08 | 172.92 | 1435.32 |
|  | ***D. rerio*** | 37,211 | 7362.78 | 1038.29 | 5.93 | 174.98 | 1281.92 |
|  | ***D. labrax*** | 46,982 | 5986.98 | 975.31 | 5.37 | 181.61 | 1146.75 |
|  | ***G. aculeatus*** | 33,218 | 7310.81 | 1101.81 | 6.58 | 167.43 | 1112.61 |
|  | ***L. crocea*** | 43,199 | 7279.58 | 1069.54 | 5.80 | 184.47 | 1294.31 |
|  | ***O. latipes*** | 32,309 | 7235.06 | 1081.32 | 6.38 | 169.54 | 1144.28 |
|  | ***T. rubripes*** | 32,269 | 7522.36 | 1099.16 | 6.61 | 166.32 | 1145.20 |
| **RNAseq** | | 14,638 | 12282.62 | 1417.98 | 10.81 | 274.74 | 949.67 |
| **IsoSeq** | | 45,439 | 13092.18 | 898.41 | 7.98 | 321.87 | 1507.56 |
| **CEGMA** | | 767 | 4912.31 | 1140.59 | 9.14 | 124.76 | 463.24 |
| **MAKER** | | 21,143 | 14003.69 | 1729.02 | 10.61 | 252.57 | 1177.61 |
| **HiCESAP** | | 22,122 | 12518.83 | 1678.18 | 10.35 | 238.02 | 1074.85 |

SI Table 10. Functional annotation of predicted protein-coding genes

|  | | Number | Percent (%) |
| --- | --- | --- | --- |
| **Total** | | 22,122 |  |
| **Annotated** | **InterPro** | 19,781 | 89.42 |
|  | **GO** | 15,101 | 68.26 |
|  | **KEGG** | 21,637 | 97.81 |
|  | **Swissprot** | 20,492 | 92.63 |
|  | **TrEMBL** | 21,713 | 98.15 |
| **Unannotated** | | 354 | 1.60 |

SI Table 11. Non-coding gene prediction in Chinese sillago genome

| Type | | Copy | Average length (bp) | Total length (bp) | % of genome |
| --- | --- | --- | --- | --- | --- |
| **miRNA** | | 283 | 83.01 | 23,492 | 0.00440 |
| **tRNA** | | 2,090 | 74.46 | 155,620 | 0.02913 |
| **rRNA** | **rRNA** | 24 | 332.54 | 7,981 | 0.00149 |
|  | **18S** | 3 | 1838.00 | 5,514 | 0.00103 |
|  | **28S** | 0 | 0.00 | 0 | 0.00000 |
|  | **5.8S** | 3 | 156.00 | 468 | 0.00009 |
|  | 5S | 18 | 111.06 | 1,999 | 0.00037 |
|  | snRNA | 255 | 134.05 | 34,182 | 0.00640 |
| snRNA | CD-box | 108 | 118.37 | 12,784 | 0.00239 |
|  | HACA-box | 61 | 144.74 | 8,829 | 0.00165 |
|  | splicing | 77 | 136.53 | 10,513 | 0.00197 |
|  | scaRNA | 9 | 228.44 | 2,056 | 0.00039 |
