# Supplementary material for: A draft genome assembly of the Chinese sillago (Sillago sinica), the first reference genome for Sillaginidae fishes
Source: Gigascience. 2018 Sep 10;7(9):giy108. doi: 10.1093/gigascience/giy108 (PMC6143730; doi:10.1093/gigascience/giy108)
Supplement: Response_to_Reviewer_Comments_Original_Submission.pdf [file giy108_response_to_reviewer_comments_original_submission.pdf]

Author's Response To Reviewer Comments

Close

Dear Editors:

We are submitting the revised manuscript entitled “A draft genome assembly of the Chinese sillago (Sillago sinica), the first reference genome for Sillaginidae fishes”. Attached please find our detailed responses to specific points raised by both reviewers. All authors greatly appreciate the feedback and we have used the suggestions to guide our revisions. We have made detailed revisions to our manuscript and have clearly answered all questions posed by the editor and the reviewers.

We believe the quality and clarity of the manuscript has improved greatly due to the feedback of the reviewers. We hope that the paper is now in a form suitable for publication in Giga Science as a Data Note.

Yours sincerely,

Prof. Dr. Tianxiang Gao
Fishery College of Zhejiang Ocean University
Zhoushan, Zhejiang, China
Email: gaotianxiang0611@163.com
March 27, 2018

Comments from the editor:
Their reports are below. Please also take a moment to check our website athttps://giga.editorialmanager.com/ to download an annotated version of the manuscript kindly provided by reviewer 1, that was saved as attachment.
Reply: We have downloaded the comments from the reviewer1 and used it to revise the manuscript according to all the suggestions.

In the revised manuscript, please also include the fishbase ID for the species. Before submitting your revised manuscript, please make sure all raw data is submitted to the SRA, with all relevant accession numbers cited in the data availability section of the paper.
Reply: We have included the fishbase ID for Sillago sinica in our manuscript. All raw data were also submit to NCBI SRA database, and the accession number was cited in this revised manuscript. (line 283-285)

Comments from the reviewers:
Reviewer reports:
Reviewer #1: The authors have produced a high quality, professional genome assembly, using PacBio and Illumina technology. The unexpected high heterozygosity of this fish makes genome assembly difficult with short reads. Use of PacBio long reads in depth produced a high quality genome, even with the awkwardness of the high heterozygosity. Minor corrections to the English and comments on parts of the manuscript are attached as a track changes word document. The annotation process is somewhat unusual, but the measure of its success lies solely with the % of full length peptides predicted. The CEGMA/BUSCO

% of full length genes needs to be added, as the authors only mention the % identified genes. This is an important and critical result for gauging the assembly quality.
Reply: We appreciate the reviewer's comments. Indeed, the high heterozygosity is one of the biggest challenge for many fish genome assembly projects. Our work, an application of PacBio sequencing to assembly fish genome with heterozygosity, could provide valuable reference for other fish genome assembly projects in the research community. We have carefully revised the manuscript according to the reviewers' suggestions for English, annotation, genome assembly evaluation, and all corrections were highlighted by red.

Below are several questions in the track changes from the reviewer 1:
how does the mapping ratio correlate with the heterozygosity measurement? Pretty well by the look.
Reply: Heterozygosity of the genome might influence the paired-end mapping ratio; however, it should be the main factor dominating the read mapping ratio. The completeness and accuracy of genome assembly are two main reasons dominating read mapping ratio.

Was Rfam searched using Infernal? This needs to be put explicitly as it could be interpreted as search by sequence similarity
Reply: This is an embarrassing error. The Rfam annotation were searched using Infernal. We corrected the method description and the revised context were highlighted in red (line 236).

Reviewer #2:
The authors report on the genome assembly of a heterozygous specimen of Sillago sinica, a species of the smelt-whiting family. This species occurs in the northwest Pacific Ocean, and was described in 2011 based on phenotypic and molecular data (COI). Sillaginidae contains several species that are difficult to distinguish morphologically. The authors use an established combination of various sequencing technologies (Illumina HiSeq and long-range PacBio) to generate several assemblies that are subsequently merged. Annotation is performed in silico based on sequence homology with other teleosts and species-specific RNA data. The authors use various methods to assess the quality of their genome assembly.

The paper is a description of the assembly and no novel biological insights are reported. Teleost genomes are notoriously difficult to assemble, often yielding highly fragmented genomes. The combined approach used here delivers a contig continuity that is in the upper range of those reported for fish, and is a clear demonstration of the value of including long-range PacBio for genome assembly. The authors claim that their contig length is remarkably larger than other teleost assemblies, but leave reasons for this (whether of technical or biological nature) unexplored. Nevertheless, this assembly remains fragmented, unordered and lacks chromosome level resolution. The concept of quality as emphasized twice in the title, is therefore ambiguous and this assembly represents a draft genome. That status should be reflected in the title. The authors should address their use of ambiguous quality statements (only x number of contigs, highly contiguous, high quality, remarkably and so forth) throughput the paper. The assembly will be of value for researchers working on Sillaginidae.
Reply: We are grateful to the reviewer for all the constructive suggestions, which help to improve the manuscript greatly. Indeed the genome assembly for the Sillago sinica remains fragmented, and we have re-titled the manuscript as "A draft genome assembly of the Chinese sillago (Sillago sinica), the first reference genome for Sillaginidae fishes" to better reflect the genome quality. For the reviewer's concern regarding to genome quality

evaluation, a separated section (Genome quality evaluation) was described to assess the assembly quality (line 168). To clarify the quality of genome assembly, we have revised the context of the assembly throughout the manuscript according to the reviewer's comments. All correction were highlighted in red (title and line 267).

Figure and table legends are not complete and absent in most cases. It should be possible to understand figures and tables without the main text.
Reply: This is embarrassing that we neglected figure and table legends. In present version, we have completed the legends for figures and tables to facilitate the reader to understand the contents. The revision were shown in red (line 310-312, 314-318, 312-313, 329-330, 332-334).

Minor:
Numerous grammatical and or spelling errors appear throughout the text.
Lines 127-128:" After removing adaptor sequences, we obtained 3.4 million subreads (totally 27.2 Gb) with a contig N50 length of 12.96kb (SI Table 3, SI Figure 3)." A read is not a contig.
Reply: We corrected the contig N50 length to read N50 length (line 148).

In my opinion, for this report it is irrelevant that this project contributes to the future Genome 10K project. If this project was funded through Genome 10K that can be mentioned elsewhere.
Reply: This work was not funded by Genome 10K project, but the data and assembly results could be useful for other genome projects, such as Genome 10K, and for genomic analysis for Sillaginidae. We have deleted the description related to the Genome 10K project in the manuscript.

Reviewer #3: The purpose of the study, High-quality genome assembly of the Chinese sillago, by Xu et al., was to assemble and annotate a long-contiguity genome of the Chinese sillago, Sillago sinica.

High molecular weight genomic DNA with fragment size around 20kb was extracted from muscle tissues. Five paired-end (PE) Illumina libraries with insert sizes 250 bp, 300 bp, 500 bp, 800 bp, and 2kb were sequenced on the Illumina HiSeq platform. The genome size was estimated using a k-mer approach. After constructing a pilot genome assembly, two additional genomic libraries with insert size 20 kb were prepared and sequenced using five SMRT cells on PacBio Sequel. The PacBio sequences were assembled, first by using FALCON and second by using Canu genome assembler. Both assemblies were merged using Genome Puzzle Master (GPM) and then the integrated assembly was polished using Illumina data to generate a final, integrated and polished, genome assembly of S. sinica. The genome quality evaluation was performed using CEGMA and BUSCO to validate the completeness of eukaryotic ortholog genes in the genome assembly.

Based on the distribution of k-mer of size 17, the genome size was estimated to be 543 Mb with 66x estimated coverage, 0.76% heterozygosity (which is higher than that of other fish species), and 12.7% repeat content. The genome assembly based only on Illumina data was of low quality with a total size of 624Mb and 3.2Kb contig N50. The Falcon/Canu hybrid genome assembly based on PacBio sequences (and polished with Illumina sequences) was 534Mb in size with a 2.6Mb contig N50, 802 contigs, and 96% complete orthologous genes. A total of 22,122 protein coding genes were annotated.

The manuscript by Xu, et al., reports a high-quality genome assembly of the Chinese sillago, the first among species within Sillaginidae. The data is solid with appropriate analyses and appropriate interpretations of the results. However, minor revisions are required.

1. Lines 61-62: Authors' claim "Owing to similar phenotypic characteristics, delineation and identification of Sillaginidae species often confuse the taxonomists" is not supported by reference.
Reply: We have added references for the sentence (line 61). Owing to similar phenotypic characteristics, many misidentified species were identified in the last decade using molecular markers. Therefore, we believe that genomic data in this work could prompt the species identification studies for the Sillaginidae in the future.

2. Lines 62-64: Authors' claim "Rapid environment changes resulted from anthropogenic activities can force Sillaginidae species adapt to diversifying situations, leading to further diversification and speciation" is not supported by reference.Lines 65-66: Authors' statement "Numerous cryptic lineages were identified in S. sihama complex by using phenotypic traits and molecular markers in the Northwestern Pacific" does not have reference to support it.
Reply: A reference was added to support the sentence. (line 64)

3. Lines 66-67: Authors stated, "five recently identified Sillago species were misidentified as S. sihama", but did not provide information on whether the method used for identification was solely phenotypic or both phenotypic or molecular. Lines 70-71. Authors stated, "among Sillaginidae species, the Chinese sillago Sillago sinica is one the most recently identified Sillaginidae species in the Northwestern Pacific", but did not provide information on the previously used method to identify S. sinica.
Reply: We appreciate the suggestions. Previous misidentification were made solely on phenotypic data. However, the application of molecular markers helped to reveal cryptic species among Sillaginidae species in recent years. We have added the methods and information used to the species identification. The revision were highlighted in red (69-71).

5. Line 72. Authors stated, "Due to their phenotypic similarity, S. sinica was previously misidentified as S. sihama", but did not support the statement by reference.
Reply: References were added for the sentence (line 74).

6. Lines 73-74. Authors stated, "two species are different because S. sinica inhabits cold-temperate environment while S. sihama inhabits warm-temperate environment", but did not provide any reference to support that the same species can't inhabit different environments.
Reply: A reference was added to support the sentence (line 75).

7. Lines 74-77. Authors concluded, "It is thus essential to sequence the genome of S. sinica", and claimed that sequencing the genome of S. sinica "will improve taxonomy, and may help to reveal insights into evolutionary history of Sillaginidae species and the role of environment changes in rapid genetic diversification and speciation". However, authors did not provide any support for their claims.
Reply: References were added to support the context. Previous studies have shown the contribution of the genome on environmental adaptation and evolutionary studies, we also cited the related researches for other fish species. (line 78)

8. Line 87. Authors stated, "we collected fresh muscle tissue", however it is not clear what

type of muscle tissue was collected (red muscle tissue or white muscle tissue?).
Reply: Epaxial white muscle tissues were collected for DNA extraction and sequencing. We have added the information into the manuscript and highlighted the revision in red (line 88).

9. Lines 91-92. Authors claimed, "a main band around 20 kb indicating high-quality for PacBio Sequel platform", but did not provide reference to support why 20 kb indicates quality. Perhaps they mean the DNA is high molecular weight, or is long enough for sequencing on the PacBio platform.
Reply: The resolution of the conventional agarose gel electrophoresis was 200 bp to 20 kb. In this work, 20 kb libraries were prepared for PacBio sequencing; therefore, a single band above 20 kb on an agarose gel indicated that lengths of DNA in the band were at least 20kb, and the integrity of DNA molecules satisfied the requirement for PacBio library construction (https://www.pacb.com/wp-content/uploads/2015/09/Guide-Pacific-Biosciences-Template-Preparation-and-Sequencing.pdf). (line 93)

10. Lines 95. Authors stated, "we also sequenced the genomic DNA using Illumina DNA sequencing technologies", but did not provide information on the amount of DNA used for sequencing.
Reply: 20 ug DNA molecules were used for library construction and Illumina DNA sequencing. We have added the information in the context (line 97-98).

Lines 96-97. Authors stated, "Five paired-end libraries were constructed with insert sizes of 250 base pairs (bp), 300 bp, 500 bp, 800 bp, 2 kb and generated a total of ~42 Gb sequence data", but did not provide the reason(s) of using different insert sizes, the technical type of libraries (was the 2 kb library a mate-pair library, or a paired-end library?), and the meaning of ~42 Gb sequence data - what coverage of the genome was generated by each library?.
Reply: We thanks reviewer for the important concern. Sequencing with multiply libraries for various insertion length was a traditional strategy for genome assembly using Illumina sequencing platform. Before the application of PacBio platform, we first tried to assess the feasibility to assembly the Chinese sillago solely using the Illumina platform, therefore, various insertion length (250 base pairs (bp), 300 bp, 500 bp, 800 bp, 2 kb) were generated. The library of 2kb was a mate-pair library. We have corrected the library type of 2kb (line 97).
We are sorry that we made a mistake to calculate the sequencing amount (~42Gb) for 300 bp library as the total NGS sequencing data. Actually, 35, 42, 31, 39 and 18 Gb data were generated, representing the genome coverage of 67X, 81X, 60X, 75X and 35X, for 250 bp, 300 bp, 500 bp, 800 bp, 2 kb, respectively, resulting into a total of 165 Gb NGS data (a coverage of ~317X). We added the information into the revised manuscript (line 98-99) and Table 1.

11. Line 99. Authors stated, "Raw reads were analyzed using FastQC and then filtered using HTQC". However, the following things are not clear: a) the kind of analysis performed by using FastQC, and b) the type of sequence filtered using HTQC.
Reply: We thank the reviewer for this reminding. FastQC was used for quality control, including the base and read quality evaluation, and HTQC was used for quality and length trimming. We have added the information in the revised manuscript (line 101-102).

12. Lines 100-105. Authors stated, "reads were filtered in the following filtering steps: 1) Removing adaptors…………..; 2) Removing read pairs……….;3) Trimming ambiguous or low quality fragments………..; 4) Removing read pairs………", but it is not clear which

software performed which step.
Reply: We thank reviewer for the reminding. All above analysis were performed with FastQC and HTQC. We have added the information in the manuscript (line 103).

13. Line 105. What does "A single peak around 45%"mean? Also, which software was used to see GC distribution?
Reply: GC distribution in reads were calculated and analyzed by FastQC. We have added the information into the manuscript (line 107-108).

14. Line 106-107. Authors stated, "After searching against to non-redundant nucleotide (nt) database with BLASTN, we found that the best hits were enriched to closely related fish species". However, authors could provide the following information: the type of sequence that was searched against non-redundant nucleotide database using BLASTN, the reason of the search, and the place where the result of this analysis could be found.
Reply: Short reads from NGS might contained the contamination during library construction and sequencing. To check if there is any obvious contamination in the sequencing data, we randomly selected 10,000 paired sequencing reads and searched against the NT database. From our result, no obvious contamination was observed. We have revised the manuscript (line 113-114) and added the table in the revised Supplementary Information Table 2.

15. Line 108-109. What are the common names of the "closely related fish species"?
Reply: We have added the common names of the closely related fish species (line 111-113).

16. Line 110. Authors stated, "We estimated the genome size of the Chinese sillago by analyzing the 17-mer depth distribution". However, it is not clear what type of reads were used for creating 17-mer distribution (raw reads or cleaned sequence reads and from which molecular libraries). Further, authors conducted K-mer based method of genome size estimation. However, there is no information on the following things: a) Why was 17-mer chosen? b) Was the process iterated for different k-mers? How was k-mer distribution generated? How was the peak position determined? Which software was used for genome size estimation based on 17-kmer? The author use an ambiguous citation (citation #16) to "gce" with no further information.
Reply: The comments from the reviewer was very important. Clean reads after quality trim were used for all sequencing analysis, including Kmer analysis. Previous studies1 often require the Kmer space to be at least 5 times larger than the genome size (4K>5*G), and the larger the better. Kmer size of 17 satisfy the requirement.
17-mer were generated using gce software1, which called jellyfish for K-mer distribution generation. The peak position was also determined by gce. We have added the reference in the revised manuscript (line 121). In addition, we updated the reference for the Kmer method for genome size estimation (line 116). We applied 17, 21 and 27 for Kmer size, and found that the estimated genome size ranged from 519 to 524 Mb. The resulted were added to the Supplementary Information (SI Table 3) and the information was added to the revised manuscript (line 123-125).

17. Line 113. Authors provided the meaning of N17-mer and D17-mer explicitly but not that of 'G'.
Reply: The G here mean the estimated genome size. We have added the information in the revised manuscript. (line 120)

18. Lines 114-115. Authors stated, "For our data, N17-mer was 37,811,957,476 and D17-

mer was 66, suggesting an estimated genome size of 524 Mb", but did not state which value suggests the estimated genome coverage.
Reply: Thanks for reviewer's comments. From the K-mer method, the peak of K-mer distribution was the estimation of the genomic sequencing coverage, which was 66 in this work (line 121-122).

19. Lines 115-116. How were "heterozygosity" and "repeat content" estimated?
Reply: Thanks for reviewer's comments. The heterozygosity and repeat content were estimated from the statistical models from the Kmer distribution. The underlying principle were comprehensively explained in the article of gce software1. We have added the information in the revised manuscript (line 126 and 127).

20. Line 120. Authors should point out what "artificial breeding" does to the genome heterozygosity.
Reply: Thanks a lot for the reviewer's comment. Many artificial breeding techniques in aquaculture, such as inbreeding and gynogenesis, could effectively minimize the genomic heterozygosity and potentially reduce the difficulty of the genome assembly. We have added reference for the genome assembly of a farmed fish species, grass carp2, in the revised manuscript (line 131-133).

21. Line 120. What were the reason for generating a "pilot assembly"?
Reply: We thank the reviewer for the important concern. As our response to the above comment, we first tried to assembly the Chinese sillago solely using the Illumina platform with various insertion length libraries, which was called the pilot assembly. The assembly from the pilot assembly was high fragmented, we therefore instead applied the PacBio platform for the genome assembly. We have revised the manuscript to clarify (line 134-145).

22. Line 121. Authors used "Platanus package" to generate "pilot genome assembly". However, it is unknow why Platanus package was used and how Platanus package was used. What parameters were employed and was another assembler used as well for comparison?
Reply: We appreciate this important concern. Genomic heterozygosity is one of the biggest challenge of many complex genome assembly.3 Platanus package were designed for heterozygous genome assembly and exhibited excellent performance in several complex genome3. Therefore, we used the package for our pilot genome assembly. The default parameters were used for the genome assembly. We did not applied other assembler for the genome since we focused on PacBio assembly in this work (line 134-139).

23. Line 122-123. Authors stated, "genome assembly was of low-quality partly due to its high genomics heterozygosity", however it is unknown why the pilot genome assembly was considered of low quality. Also, assuming by "low-quality" the authors are referring to contig N50, a low value here is much more likely to be attributed to the insert sizes of the molecular library (maximum size 2 kb) as opposed to heterozygosity.
Reply: The comments helped us to improve the context from line 137 to 142. The assembly solely using NGS data was highly fragmented and the low-quality here was referred to the continuity, namely contig N50. We have revised the manuscript and deleted "low-quality" and its ambiguous correlation to"high genomics heterozygosity".

24. Line 125-126. Authors stated, "We prepared two 20 kb genomic DNA libraries, which we sequenced using PacBio Sequel using five SMRT cells, generating 27.3 Gb raw reads",

but the reason of using long read sequences to assemble the genome is not mentioned. Again, the meaning of 27.3 Gb raw DNA reads is unknown, what coverage was generated?
Reply: Because of short reads, the assembly using traditional NGS short sequencing data resulted into highly fragmented genome for Chinese sillago in this work. Previous studies illuminate the excellent performance of PacBio long reads on complex genome assembly4,5; we therefore applied PacBio to generate long reads, aiming to generate longer contig assembly for the genome. The PacBio sequencing data of 27.3 Gb represented the ~53X coverage of the genome. We have added the information in the revised manuscript (line 148).

25. SI Figure 2. On graph authors could indicate the portions of the graph that show error k-mers, true k-mers, peak by heterozygotic alleles, main peak, and repeats.
Reply: We have revised SI Figure 2 to indicate the error k-mers, true k-mers, peak by heterozygotic alleles, main peak, and repeats.

26. SI Figure 4. Genome sequence validation using NGS reads from the libraries with various length was performed and the result is shown in SI Figure 4 but nothing is presented on text about it.
Reply: We have clarified the context of SI Figure 4 in the line 179-181 in the revised manuscript, as a supporting evidence of genome quality.

27. The text needs to be copy-edited to fix a number of simple English grammatical errors.
Reply: We have carefully revised the English grammatical errors through the manuscript. All corrections were highlighted in red.

References

1 Liu, B. et al. Estimation of genomic characteristics by analyzing k-mer frequency in de novo genome projects. Quantitative Biology 35, 62-67 (2013).
2 Wang, Y. et al. The draft genome of the grass carp (Ctenopharyngodon idellus) provides insights into its evolution and vegetarian adaptation. Nature Genetics 47, 625-631 (2015).
3 Kajitani, R. et al. Efficient de novo assembly of highly heterozygous genomes from whole-genome shotgun short reads. Genome Research 24, 1384-1395 (2014).
4 Conte, M. A., Gammerdinger, W. J., Bartie, K. L., Penman, D. J. & Kocher, T. D. A high quality assembly of the Nile Tilapia (Oreochromis niloticus) genome reveals the structure of two sex determination regions. Bmc Genomics 18, 341 (2017).
5 Fu, X. et al. Long-read sequence assembly of the firefly Pyrocoelia pectoralis genome. Gigascience 6, 1-7 (2017).

Close
