# Supplementary material for: A draft genome assembly of the Chinese sillago (Sillago sinica), the first reference genome for Sillaginidae fishes
Source: Gigascience. 2018 Sep 10;7(9):giy108. doi: 10.1093/gigascience/giy108 (PMC6143730; doi:10.1093/gigascience/giy108)
Supplement: Reviewer_2_Report_(Original_Submission) -- Bastiaan Star — 05/14/2018 Reviewed [file giy108_reviewer_2_report_(original_submission).pdf]

Reviewer Report

Title: **A draft genome assembly of the Chinese sillago (Sillago sinica), the first reference genome for Sillaginidae fishes**

Version: **Original Submission** **Date:** 05/14/2018

Reviewer name: **Bastiaan Star**

Reviewer Comments to Author:

The authors report on the genome assembly of a heterozygous specimen of Sillago sinica, a species of the smelt-whiting family. This species occurs in the northwest Pacific Ocean, and was described in 2011 based on phenotypic and molecular data (COI). Sillaginidae contains several species that are difficult to distinguish morphologically. The authors use an established combination of various sequencing technologies (Illumina HiSeq and long-range PacBio) to generate several assemblies that are subsequently merged. Annotation is performed in silico based on sequence homology with other teleosts and species-specific RNA data. The authors use various methods to assess the quality of their genome assembly.
The authors report on the genome assembly of a heterozygous specimen of Sillago sinica, a species of the smelt-whiting family. This species occurs in the northwest Pacific Ocean, and was described in 2011 based on phenotypic and molecular data (COI). Sillaginidae contains several species that are difficult to distinguish morphologically. The authors use an established combination of various sequencing technologies (Illumina HiSeq and long-range PacBio) to generate several assemblies that are subsequently merged. Annotation is performed in silico based on sequence homology with other teleosts and species-specific RNA data. The authors use various methods to assess the quality of their genome assembly.

The paper is a description of the assembly and no novel biological insights are reported. Teleost genomes are notoriously difficult to assemble, often yielding highly fragmented genomes. The combined approach used here delivers a contig continuity that is in the upper range of those reported for fish, and is a clear demonstration of the value of including long-range PacBio for genome assembly. The authors claim that their contig length is remarkably larger than other teleost assemblies, but leave reasons for this (whether of technical or biological nature) unexplored. Nevertheless, this assembly remains fragmented, unordered and lacks chromosome level resolution. The concept of quality as emphasized twice in the title, is therefore ambiguous and this assembly represents a draft genome. That status should be reflected in the title. The authors should address their use of ambiguous quality statements (only x number of contigs, highly contiguous, high quality, remarkably and so forth) throughput the paper. The assembly will be of value for researchers working on Sillaginidae.

Figure and table legends are not complete and absent in most cases. It should be possible to understand figures and tables without the main text.

Minor:
Numerous grammatical and or spelling errors appear throughout the text.

Lines 127-128:" After removing adaptor sequences, we obtained 3.4 million subreads (totally 27.2 Gb) with a contig N50 length of 12.96kb (SI Table 3, SI Figure 3)." A read is not a contig.

In my opinion, for this report it is irrelevant that this project contributes to the future Genome 10K project. If this project was funded through Genome 10K that can be mentioned elsewhere. **Level of Interest**

Please indicate how interesting you found the manuscript: An article of limited interest

Quality of Written English

Please indicate the quality of language in the manuscript: Acceptable

Declaration of Competing Interests

Please complete a declaration of competing interests, considering the following questions:

- Have you in the past five years received reimbursements, fees, funding, or salary from an organisation that may in any way gain or lose financially from the publication of this manuscript, either now or in the future?
- Do you hold any stocks or shares in an organisation that may in any way gain or lose financially from the publication of this manuscript, either now or in the future?
- Do you hold or are you currently applying for any patents relating to the content of the manuscript?
- Have you received reimbursements, fees, funding, or salary from an organization that holds or has applied for patents relating to the content of the manuscript?
- Do you have any other financial competing interests?
- Do you have any non-financial competing interests in relation to this paper?

If you can answer no to all of the above, write 'I declare that I have no competing interests' below. If your reply is yes to any, please give details below.

I declare that I have no competing interests

I agree to the open peer review policy of the journal. I understand that my name will be included on my report to the authors and, if the manuscript is accepted for publication, my named report including any attachments I upload will be posted on the website along with the authors' responses. I agree for my report to be made available under an Open Access Creative Commons CC-BY license (http://creativecommons.org/licenses/by/4.0/). I understand that any comments which I do not wish to be included in my named report can be included as confidential comments to the editors, which will not be published.

I agree to the open peer review policy of the journal

To further support our reviewers, we have joined with Publons, where you can gain additional credit to further highlight your hard work (see: https://publons.com/journal/530/gigascience). On publication of this paper, your review will be automatically added to Publons, you can then choose whether or not to claim your Publons credit. I understand this statement. Yes
