# Supplementary material for: A draft genome assembly of the Chinese sillago (Sillago sinica), the first reference genome for Sillaginidae fishes
Source: Gigascience. 2018 Sep 10;7(9):giy108. doi: 10.1093/gigascience/giy108 (PMC6143730; doi:10.1093/gigascience/giy108)
Supplement: Reviewer_3_Report_(Original_Submission) -- Julian Catchen — 05/31/2018 Reviewed [file giy108_reviewer_3_report_(original_submission).pdf]

Reviewer Report

Title: **A draft genome assembly of the Chinese sillago (Sillago sinica), the first reference genome for Sillaginidae fishes**

Version: **Original Submission** **Date:** 05/31/2018

Reviewer name: **Julian Catchen**

Reviewer Comments to Author:

The purpose of the study, High-quality genome assembly of the Chinese sillago, by Xu et al., was to assemble and annotate a long-contiguity genome of the Chinese sillago, Sillago sinica.

High molecular weight genomic DNA with fragment size around 20kb was extracted from muscle tissues. Five paired-end (PE) Illumina libraries with insert sizes 250 bp, 300 bp, 500 bp, 800 bp, and 2kb were sequenced on the Illumina HiSeq platform. The genome size was estimated using a k-mer approach. After constructing a pilot genome assembly, two additional genomic libraries with insert size 20 kb were prepared and sequenced using five SMRT cells on PacBio Sequel. The PacBio sequences were assembled, first by using FALCON and second by using Canu genome assembler. Both assemblies were merged using Genome Puzzle Master (GPM) and then the integrated assembly was polished using Illumina data to generate a final, integrated and polished, genome assembly of S. sinica. The genome quality evaluation was performed using CEGMA and BUSCO to validate the completeness of eukaryotic ortholog genes in the genome assembly.

Based on the distribution of k-mer of size 17, the genome size was estimated to be 543 Mb with 66x estimated coverage, 0.76% heterozygosity (which is higher than that of other fish species), and 12.7% repeat content. The genome assembly based only on Illumina data was of low quality with a total size of 624Mb and 3.2Kb contig N50. The Falcon/Canu hybrid genome assembly based on PacBio sequences (and polished with Illumina sequences) was 534Mb in size with a 2.6Mb contig N50, 802 contigs, and 96% complete orthologous genes. A total of 22,122 protein coding genes were annotated.

The manuscript by Xu, et al., reports a high-quality genome assembly of the Chinese sillago, the first among species within Sillaginidae. The data is solid with appropriate analyses and appropriate interpretations of the results. However, minor revisions are required.

1. Lines 61-62: Authors' claim "Owing to similar phenotypic characteristics, delineation and identification of Sillaginidae species often confuse the taxonomists" is not supported by reference.
2. Lines 62-64: Authors' claim "Rapid environment changes resulted from anthropogenic activities can force Sillaginidae species adapt to diversifying situations, leading to further diversification and speciation" is not supported by reference.
3. Lines 65-66: Authors' statement "Numerous cryptic lineages were identified in S. sihama complex by using phenotypic traits and molecular markers in the Northwestern Pacific" does not have reference to support it.
Lines 66-67: Authors stated, "five recently identified Sillago species were misidentified as S. sihama", but did not provide information on whether the method used for identification was solely phenotypic or both phenotypic or molecular.
4. Lines 70-71. Authors stated, "among Sillaginidae species, the Chinese sillago Sillago sinica is one the most recently identified Sillaginidae species in the Northwestern Pacific", but did not provide information on the previously used method to identify S. sinica.
5. Line 72. Authors stated, "Due to their phenotypic similarity, S. sinica was previously misidentified as S. sihama", but did not support the statement by reference.
6. Lines 73-74. Authors stated, "two species are different because S. sinica inhabits cold-temperate environment while S. sihama inhabits warm-temperate environment", but did not provide any reference to

support that the same species can't inhabit different environments.
7. Lines 74-77. Authors concluded, "It is thus essential to sequence the genome of S. sinica", and claimed that sequencing the genome of S. sinica "will improve taxonomy, and may help to reveal insights into evolutionary history of Sillaginidae species and the role of environment changes in rapid genetic diversification and speciation". However, authors did not provide any support for their claims.
8. Line 87. Authors stated, "we collected fresh muscle tissue", however it is not clear what type of muscle tissue was collected (red muscle tissue or white muscle tissue?).
9. Lines 91-92. Authors claimed, "a main band around 20 kb indicating high-quality for PacBio Sequel platform", but did not provide reference to support why 20 kb indicates quality. Perhaps they mean the DNA is high molecular weight, or is long enough for sequencing on the PacBio platform.
10. Lines 95. Authors stated, "we also sequenced the genomic DNA using Illumina DNA sequencing technologies", but did not provide information on the amount of DNA used for sequencing.
Lines 96-97. Authors stated, "Five paired-end libraries were constructed with insert sizes of 250 base pairs (bp), 300 bp, 500 bp, 800 bp, 2 kb and generated a total of ~42 Gb sequence data", but did not provide the reason(s) of using different insert sizes, the technical type of libraries (was the 2 kb library a mate-pair library, or a paired-end library?), and the meaning of ~42 Gb sequence data - what coverage of the genome was generated by each library?.
11. Line 99. Authors stated, "Raw reads were analyzed using FastQC and then filtered using HTQC". However, the following things are not clear: a) the kind of analysis performed by using FastQC, and b) the type of sequence filtered using HTQC.
12. Lines 100-105. Authors stated, "reads were filtered in the following filtering steps: 1) Removing adaptors…………..; 2) Removing read pairs……….;3) Trimming ambiguous or low quality fragments………..; 4) Removing read pairs………", but it is not clear which software performed which step.
13. Line 105. What does "A single peak around 45%"mean? Also, which software was used to see GC distribution?
14. Line 106-107. Authors stated, "After searching against to non-redundant nucleotide (nt) database with BLASTN, we found that the best hits were enriched to closely related fish species". However, authors could provide the following information: the type of sequence that was searched against non-redundant nucleotide database using BLASTN, the reason of the search, and the place where the result of this analysis could be found.
15. Line 108-109. What are the common names of the "closely related fish species"?
16. Line 110. Authors stated, "We estimated the genome size of the Chinese sillago by analyzing the 17-mer depth distribution". However, it is not clear what type of reads were used for creating 17-mer distribution (raw reads or cleaned sequence reads and from which molecular libraries). Further, authors conducted K-mer based method of genome size estimation. However, there is no information on the following things: a) Why was 17-mer chosen? b) Was the process iterated for different k-mers? How was k-mer distribution generated? How was the peak position determined? Which software was used for genome size estimation based on 17-kmer? The author use an ambiguous citation (citation #16) to "gce" with no further information.
17. Line 113. Authors provided the meaning of N17-mer and D17-mer explicitly but not that of 'G'.
18. Lines 114-115. Authors stated, "For our data, N17-mer was 37,811,957,476 and D17-mer was 66, suggesting an estimated genome size of 524 Mb", but did not state which value suggests the estimated genome coverage.
19. Lines 115-116. How were "heterozygosity" and "repeat content" estimated?
20. Line 120. Authors should point out what "artificial breeding" does to the genome heterozygosity.
21. Line 120. What were the reason for generating a "pilot assembly"?
22. Line 121. Authors used "Platanus package" to generate "pilot genome assembly". However, it is unknow why Platanus package was used and how Platanus package was used. What parameters were employed and was another assembler used as well for comparison?
23. Line 122-123. Authors stated, "genome assembly was of low-quality partly due to its high genomics heterozygosity", however it is unknown why the pilot genome assembly was considered of low quality. Also, assuming by "low-quality" the authors are referring to contig N50, a low value here is much more likely to be attributed to the insert sizes of the molecular library (maximum size 2 kb) as opposed to heterozygosity.
24. Line 125-126. Authors stated, "We prepared two 20 kb genomic DNA libraries, which we sequenced

using PacBio Sequel using five SMRT cells, generating 27.3 Gb raw reads", but the reason of using long read sequences to assemble the genome is not mentioned. Again, the meaning of 27.3 Gb raw DNA reads is unknown, what coverage was generated?
25. SI Figure 2. On graph authors could indicate the portions of the graph that show error k-mers, true k-mers, peak by heterozygotic alleles, main peak, and repeats.
26. SI Figure 4. Genome sequence validation using NGS reads from the libraries with various length was performed and the result is shown in SI Figure 4 but nothing is presented on text about it.
27. The text needs to be copy-edited to fix a number of simple English grammatical errors.

Level of Interest

Please indicate how interesting you found the manuscript: An article of limited interest

Quality of Written English

Please indicate the quality of language in the manuscript: Acceptable

Declaration of Competing Interests

Please complete a declaration of competing interests, considering the following questions:

- Have you in the past five years received reimbursements, fees, funding, or salary from an organisation that may in any way gain or lose financially from the publication of this manuscript, either now or in the future?
- Do you hold any stocks or shares in an organisation that may in any way gain or lose financially from the publication of this manuscript, either now or in the future?
- Do you hold or are you currently applying for any patents relating to the content of the manuscript?
- Have you received reimbursements, fees, funding, or salary from an organization that holds or has applied for patents relating to the content of the manuscript?
- Do you have any other financial competing interests?
- Do you have any non-financial competing interests in relation to this paper?

If you can answer no to all of the above, write 'I declare that I have no competing interests' below. If your reply is yes to any, please give details below.

I declare that I have no competing interests

I agree to the open peer review policy of the journal. I understand that my name will be included on my report to the authors and, if the manuscript is accepted for publication, my named report including any attachments I upload will be posted on the website along with the authors' responses. I agree for my report to be made available under an Open Access Creative Commons CC-BY license (http://creativecommons.org/licenses/by/4.0/). I understand that any comments which I do not wish to be included in my named report can be included as confidential comments to the editors, which will not be published.

I agree to the open peer review policy of the journal

To further support our reviewers, we have joined with Publons, where you can gain additional credit to further highlight your hard work (see: https://publons.com/journal/530/gigascience). On publication of this paper, your review will be automatically added to Publons, you can then choose whether or not to claim your Publons credit. I understand this statement. Yes
